# A melting pot of Roman dogs north of the Alps with high phenotypic and genetic diversity and similar diets

**DOI:** 10.1038/s41598-023-44060-3

**Published:** 2023-10-13

**Authors:** José Granado, Julian Susat, Claudia Gerling, Monika Schernig-Mráz, Angela Schlumbaum, Sabine Deschler-Erb, Ben Krause-Kyora

**Affiliations:** 1 Department Environmental Science, Integrative Prehistory and Archaeological Science (IPAS), University of Basel, Spalenring 145, 4055 Basel, Switzerland; 2 Institute of Clinical Molecular Biology (IKMB), Christian-Albrechts-University Kiel, Rosalind-Franklin-Strasse 12, 24105 Kiel, Germany

**Keywords:** Genetics, Molecular biology, Zoology

## Abstract

Several dog skeletons were excavated at the Roman town of Augusta Raurica and at the military camp of Vindonissa, located in the northern Alpine region of Switzerland (Germania Superior). The relationships between them and the people, the nature of their lives, and the circumstances of their deaths are unclear. In order to gain insight into this dog population, we collected 31 dogs deposited almost simultaneously in two wells (second half of the third century CE), three dogs from burial contexts (70–200 CE and third to fifth century CE) at Augusta Raurica, and two dogs from burial contexts at Vindonissa (ca. first century CE). We detected a mixed population of young and adult dogs including small, medium and large sized individuals. Three small dogs had conspicuous phenotypes: abnormally short legs, and one with a brachycephalic skull. Stable isotope analysis of a subset of the dogs showed that their diets were omnivorous with a substantial input of animal proteins and little variation, except one with a particularly low δ^15^N value, indicating a diet low in animal proteins. Partial mitochondrial DNA sequences from 25 dogs revealed eight haplotypes within canine haplogroup A (11 dogs; 44%; 5 haplotypes), C (8 dogs; 32%; 1 haplotype), D (4 dogs, 16%; 1 haplotype) and B (2 dogs, 8%; 1 haplotype). Based on shotgun sequencing, four Roman mitogenomes were assembled, representing sub-haplogroups A1b3, A1b2 and C2. No canine pathogens were identified, weakening the assumption of infectious disease as a cause for dog disposal. The genetic and morphological diversity observed in dogs of Augusta Raurica and Vindonissa is similar to modern dog diversity.

## Introduction

Since their domestication before the emergence of Neolithic farming, dogs diversified in appearance, behaviour and function, a process still ongoing^1–8^. While accompanying people across the world a close relationship was established and as consequence of their various exploitations by humans, dogs gained the ability to digest starchy food and underwent bodily changes, e.g. coat colour and floppy ears^9^.

**Figure 1:**
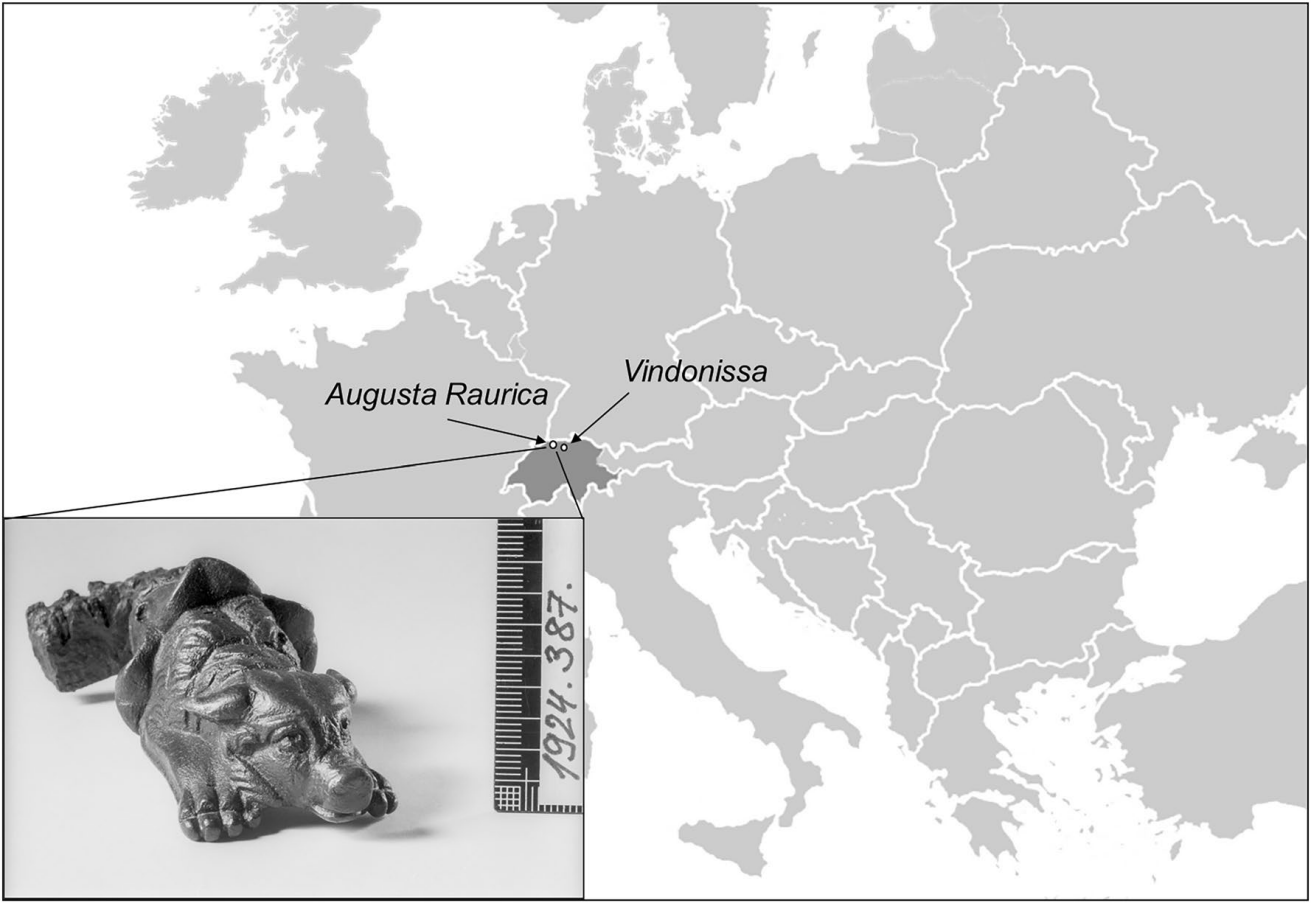
Map with approximate location of sites in Switzerland. Free available map downloaded from justfreeslide.com (https://justfreeslide.com/download/editable-europe-map-for-powerpoint/) and edited using Microsoft Office 2016. Dog figurine: Bronze key handle of a dozing dog from Augusta Raurica. Courtesy of Augusta Raurica.

Although various body sizes and coat colours are already attested for in the Magdalenian time period (18′000–12′000 BCE) and throughout prehistory^10^, in Europe dogs increasingly diversified during the Roman period, a process which likely began around the 3rd century BCE in Italy. This is well documented in texts, depicted in mosaics, and supported by objects (see Fig. 1) and archaeology^11–19^. During the Roman period dogs were particularly esteemed as pets, with a number of small dogs appearing in the Roman homeland and provinces^16,20^. For example, small-sized dogs with a brachycephalic skull (similar to Pekingese) and/or with abnormally short limbs (similar to Dachshund, Basset Hound), have been described in the archaeozoological record from a variety of Roman sites in Britannia^17,21,22^, Hispania^16,23^, Italia^24^ and Pannonia^25^. Together, the data suggest dog breeding for the purpose of selection for a specific phenotype, such as size, shortened limbs or slenderness.

Normally dogs were not part of the human diet in the Roman period and were not slaughtered^26^. In some cases, their dead bodies were even carefully buried, for example, at the Roman pet cemetery in Berenice, Egypt^27^. However, more often their bodies were deposited in rubbish pits or in wells out of use^28,29^. However, dog health, feeding and welfare issues and their complex relationship to people have rarely been addressed or brought into focus^30,31^.

In addition to archaeozoology, stable isotopes and ancient DNA offer further perspectives for addressing social relationships between humans and dogs. Stable isotope ratios in skeletal remains reflect dietary compositions^32^ and have been widely used to study faunal dietary diversity^33^. Therefore, stable isotope analysis of dog bones may shed light on their diet and living conditions. Roman authors actually recommended specific diets depending on dogs’ tasks and life stage and presumably the composition of dog food in Roman times may have varied due to the individual feeding preferences of dog owners^31^.

Genetically dogs are highly diverse. Based on mitochondrial DNA (mtDNA), European dogs belong to one of the four major haplogroups A-D^34–36^ with a dominance of A lineages in modern times, in contrast to a dominance of haplogroup C lineages during the Neolithic, suggesting a prehistoric turnover (introductions/replacements) of mtDNA lineages in Europe^1,4,37^. However, haplogroup A lineages have continuously dominated in Iberia since the Mesolithic and other lineages in central Europe have also likely survived since the Neolithic^3,19,38^. For the Roman era genetic information about dogs is scarce. Only two genetic studies addressing Roman dogs mtDNA diversity in Iberia/Morocco^20^ and Bulgaria^5^ are currently available. Their results show high mtDNA diversity with uneven geographic distribution of haplotypes belonging to the major dog haplogroups A and D (Iberia) and A, B and D (Bulgaria), denoting different histories of maternal dog lineages between the different regions of the western and eastern part of the Roman Empire, and possibly suggesting diverse centers of dog “breeding” activities.

With the northward expansion of the Roman Empire beyond the Alps and the establishment of the imperial province Germania Superior, economic, cultural and military centers such as the colonial town of Augusta Raurica and the military camp of Vindonissa in the north of Switzerland were founded^39,40^ (see also Supplementary Information). Large socio-economical changes occurred such as the introduction of horticulture, new crops and spices, new domestic animals and new technologies to Switzerland. The region was prospering and trading intensified^41^. People were very mobile in the Roman Empire^42,43^ and they transported not only objects related to daily life but they were also accompanied by their animals such as dogs. Therefore, the region considered here probably became a melting pot not only for people but also for animals. In the 2nd half of the third century CE, while the once prosperous city Augusta Raurica gradually deteriorated, several dogs were deposited in different wells, a common praxis of animal disposal during the Roman period in Europe^28,29,44^. In the case of the wells of Augusta Raurica, however, the archaeological evidence points to quick depositions within a few years or even within a few months suggesting that the animals were living at the same time and deposited almost simultaneously. Potential circumstances accompanying this event are still under debate^39,44–48^. The dogs from Vindonissa were from burial contexts and date to the 1st century CE.

This research combined osteometry, mitochondrial DNA D-loop typing, mitogenome analysis, shotgun sequencing to screen for pathogens and stable isotope analyses of dogs from Augusta Raurica and Vindonissa. The aim was to gain insight into the size, age and skeletal particularities, into the genetic variation in terms of mtDNA haplogroups and haplotypes and potential diseases as well as the diet of dogs from a local and near contemporaneous context of two wells at Augusta Raurica. The study was extended to include dogs from different time periods of Augusta Raurica and Vindonissa (Fig. 1).

## Material and methods

### Study sites and archaeological material

Samples from 36 almost complete individual Roman dog skeletons were used in this study. They originate from the town of Augusta Raurica (n = 34) and from the legionary camp of Vindonissa (n = 2) and were made available for examination by the archaeological services of Augusta Raurica and the canton of Aargau. Both sites are located in the northern Alpine margins of the Roman Imperium (today Switzerland) (Fig. 1; Table 1, Supplementary Information; Table S1). Most of the specimens from Augusta Raurica (n = 31) were discovered in two wells, namely well MR 12 ("Sodbrunnen MR 12") located in the Lower Town and well house in Insula 8 ("Brunnenhaus") in the Upper Town, filled with animal remains of different wild and domestic species (e.g., horses, pigs, bears) a custom only observed in the 2nd half of the third century CE at this site and likely coinciding with the urban decline of the city^44,47^. Numerous artefacts were also found in the wells, such as short term dated coin casting molds and pottery. Matching ceramic fragments were found scattered throughout the wells, proving that the filling is to be considered as a unit and that it took place in a short time or even in one event^44^ and for both wells at the same time span. This is also supported by the nearly completeness of the animal skeletons (Fig. S1).

**Table 1:** Information on dog specimens, sizes, age and haplogroup/haplotype belonging.

ID	Archaeological site	Type of site	Archaeological structure	Archaeological context dating	Skeletal element	Withers height (cm)	Size category	Age	Haplogroup^b)^	Haplotype (referred to as, this study)
HAL1	Augusta Raurica	Colonia, trading	Well "Sodbrunnen MR 12"	2nd half of third century CE	Femur sin	57.8	Medium	Adult	A	RS-a1
HAL2	Augusta Raurica	Colonia, trading	Well "Sodbrunnen MR 12"	2nd half of third century CE	Femur sin	41.6	Medium	Juvenile	A	RS-a3
HAL3	Augusta Raurica	Colonia, trading	Well "Sodbrunnen MR 12"	2nd half of third century CE	Femur sin	46.1	Medium	Adult	D	RS-d1
HAL4	Augusta Raurica	Colonia, trading	Well "Sodbrunnen MR 12"	2nd half of third century CE	Femur sin	50	Medium	Adult	A	RS-a4
HAL5	Augusta Raurica	Colonia, trading	Well "Sodbrunnen MR 12"	2nd half of third century CE	Femur sin	51.5	Medium	Adult	C	RS-c1
HAL6	Augusta Raurica	Colonia, trading	Well "Sodbrunnen MR 12"	2nd half of third century CE	Femur sin	54.1	Medium	Adult	D	RS-d1
HAL7	Augusta Raurica	Colonia, trading	Well "Sodbrunnen MR 12"	2nd half of third century CE	Femur dext	33.3	Small	Juvenile	D	RS-d1
HAL8	Augusta Raurica	Colonia, trading	Well "Sodbrunnen MR 12"	2nd half of third century CE	Femur sin	47.8	Medium	Adult	A	RS-a3
HAL9	Augusta Raurica	Colonia, trading	Well "Sodbrunnen MR 12"	2nd half of third century CE	Humerus sin	66.5	Large	Adult	Failed	
HAL10	Augusta Raurica	Colonia, trading	Well "Sodbrunnen MR 12"	2nd half of third century CE	Humerus dext^a)^	29.4	Small	Adult	A	RS-a2
HAL11	Augusta Raurica	Colonia, trading	Well "Sodbrunnen MR 12"	2nd half of third century CE	Femur sin	41.9	Medium	Juvenile	C	RS-c1
HAL12	Augusta Raurica	Colonia, trading	Well "Sodbrunnen MR 12"	2nd half of third century CE	Femur sin	53.1	Medium	Adult	B	RS-b1
HAL13	Augusta Raurica	Colonia, trading	Well "Sodbrunnen MR 12"	2nd half of third century CE	Humerus dext	41	Medium	Adult	A	RS-a2
HAL14	Augusta Raurica	Colonia, trading	Well "Sodbrunnen MR 12"	2nd half of third century CE	Humerus sin	63	Large	Adult	C	RS-c1
HAL15	Augusta Raurica	Colonia, trading	Well "Sodbrunnen MR 12"	2nd half of third century CE	Femurs sin	54	Medium	Adult	C	RS-c1
HAL16	Augusta Raurica	Colonia, trading	Well "Sodbrunnen MR 12"	2nd half of third century CE	Humerus sin^a)^	27	Small	Adult	A	RS-a2
HAL17	Augusta Raurica	Colonia, trading	Pit G14	Late 3rd to first half of fifth century CE	Metacarpus 4 sin	49.1	Medium	Juvenile	Not tested	
HAU1	Augusta Raurica	Colonia, trading	Well "Brunnenhaus"	2nd half of third century CE	Mandibula sin	Indeterminably	Indeterminably	Adult	C	RS-c1
HAU2	Augusta Raurica	Colonia, trading	Well "Brunnenhaus"	2nd half of third century CE	Mandibula dext	39.1	Small	Adult	failed	
HAU3	Augusta Raurica	Colonia, trading	Well "Brunnenhaus"	2nd half of third century CE	Mandibula dext	Indeterminably	Indeterminably	Juvenile	A	RS-a3
HAU4	Augusta Raurica	Colonia, trading	Well "Brunnenhaus"	2nd half of third century CE	Mandibula dext	Indeterminably	Indeterminably	Juvenile	C	RS-c1
HAU5	Augusta Raurica	Colonia, trading	Well "Brunnenhaus"	2nd half of third century CE	Metacarpus 3 sin	65.9	Large	Adult	A	RS-a1
HAU6	Augusta Raurica	Colonia, trading	Well "Brunnenhaus"	2nd half of third century CE	Femur sin	35.4	Small	Juvenile	A	RS-a2
HAU7	Augusta Raurica	Colonia, trading	Well "Brunnenhaus"	2nd half of third century CE	Humerus dext	46.8	Medium	Juvenile	A	RS-a5
HAU8	Augusta Raurica	Colonia, trading	Well "Brunnenhaus"	2nd half of third century CE	Humerus sin	53.7	Medium	Adult	B	RS-b1
HAU9	Augusta Raurica	Colonia, trading	Insula 27, hypocaust room	ca. 200 CE	Ulna dext	58	Medium	Adult	A	RS-a4
HAU10	Augusta Raurica	Colonia, trading	Well "Brunnenhaus"	2nd half of third century CE	Femur dext	62.8	Large	Adult	C	RS-c1
HAU11	Augusta Raurica	Colonia, trading	Well "Brunnenhaus"	2nd half of third century CE	Humerus dext	39.6	Small	Adult	D	RS-d1
HAU12	Augusta Raurica	Colonia, trading	Well "Brunnenhaus"	2nd half of third century CE	Radius dext	32.1	Small	Adult	Not tested	
HAU13	Augusta Raurica	Colonia, trading	Well "Brunnenhaus"	2nd half of third century CE	Femur sin	22.7	Small	Adult	Not tested	
HAU14	Augusta Raurica	Colonia, trading	Well "Brunnenhaus"	2nd half of third century CE	Femur sin	18.8	Small	Adult	Not tested	
HAU15	Augusta Raurica	Colonia, trading	Well "Brunnenhaus"	2nd half of third century CE	Metacarpus 4 dext	51.9	Medium	Adult	Not tested	
HAU16	Augusta Raurica	Colonia, trading	Well "Brunnenhaus"	2nd half of third century CE	Metatarsus 2 sin	76.2	Large	Adult	Not tested	
HAU17	Augusta Raurica	Colonia, trading	Well "Brunnenhaus"	2nd half of third century CE	Metatarsus 4 sin	64.9	Large	Adult	Not tested	
HAU18	Augusta Raurica	Colonia, trading	Pit 3 and 5 (Phase B4)	70–100 CE	Calcaneus sin	55.7	Medium	Adult	Not tested	
HAV1	Vindonissa	Military camp, cemetery	Burial	2nd third of first century CE	Tibia dext	31.7	Small	Juvenile	Failed	
HAV2	Vindonissa	Military camp	Depositing close to neonate burial	3rd third of first century CE	Radius sin	32	Small	Adult	C	RS-c1

See Table S1 for more details.

^a^All skeletal elements belong to individual dogs except humerus dext. (HAL10) and humerus sin. (HAL16) which belong to the same individual; referred to as dog HAL16/HAL10.

^b^Haplogroup assignment according to Dulbeba et al. 2015 based on diagnostic sites within D-loop region.

Additionally, two individuals were from pits and one from a hypocaust room in the residential area of Augusta Raurica^49–51^ (Table 1, Table S1). One of the dogs from Vindonissa was found in a cemetery^52^, the other (headless) dog within an officer’s house close to two neonate burials^40^ (Table 1, Table S1). Only samples from distinguishable dog individuals were used in this study.

### Metrics and morphology

Osteological analyses were carried out at IPAS, Basel, Switzerland. Withers height calculations (cm) were attempted for all individuals according to Clark^53^ and Koudelka^54^ and particular morphological features of limb bones and, in few cases skulls were also recorded (see Supplementary Information).

### Stable isotope analysis

Stable isotope analyses were carried out at IPAS, Basel, Switzerland. Stable carbon and nitrogen isotope analysis was performed on 18 dog individuals from Augusta Raurica, of which 9 were also analysed for aDNA. The same bone specimens were used for both analyses (Table S1). Analytical protocols followed Longin^55^ with modifications as described in^56^ (for more details see Supplementary Material).

### Ancient DNA methods

All aDNA data of the bones were produced independently in Basel, Switzerland and Kiel, Germany.

### Authentication of aDNA and mtDNA analysis at Basel University

To prevent contaminations, handling and processing of ancient samples followed strict standards in aDNA research established at Integrative Prehistory and Archaeological Science (IPAS)^57–59^. For details see Supplementary Information.

A 97-bp diagnostic fragment of the dog mitochondrial D-loop^1^ was PCR-amplified and sequenced to determine mtDNA haplotypes in 28 dog individuals (Table 1, Supplementary Methods, Table S1). To study relatedness in a historical context, obtained sequences were compared to published Eurasian modern, Roman and pre-Roman dog sequences with known haplogroup affiliations by using Median-Joining Network (MJN) analysis (Supplementary Methods, Table S2). Genetic diversity for 23 dogs from Augusta Raurica was calculated with Arlequin 10.0^60^ and haplogroup frequencies for Roman and pre-Roman periods and for modern dog breeds were estimated from published data (Table S2, Supplementary Information).

### Sample processing and aDNA analysis at Kiel University

Sample processing and DNA analysis were performed in clean room facilities dedicated to aDNA research at Kiel University and following the guidelines on contamination control in aDNA studies^61–63^ as described in Krause-Kyora et al.^64^. Dog samples from Augusta Raurica were pre-screened for pathogens using a shotgun sequencing approach (Table S3). Four complete dog mitochondrial genomes were assembled. A maximum likelihood tree was generated and visualized by using FigTree (Supplementary Information). Haplogroup/type assignment was according to Duleba et al.^36^.

## Results

### Morphometrics and morphology

Dog withers heights were calculated from different complete elements: femur, metapodia, humerus, tibia, ulna, calcaneus and radius. For 33 out of 36 dogs analyzed, representing 27 full-grown adult and 9 juvenile dogs highly variable sizes were obtained (Table 1, Table S1, Fig. S2). Three dogs were only represented by fragments; therefore, no withers heights could be calculated. The smallest dog had a withers height of 19 cm and the tallest reached a size of 76 cm. Adult dogs were either small (< 39 cm, n = 7), medium-sized (40–59 cm, n = 13) or large (> 60 cm, n = 6). The data show features of a morphologically varied and age-mixed population with slight differences in size distribution between wells: more medium-sized adult dogs were found in well MR 12 (“Sodbrunnen MR 12”) (n = 9, out of 12 adults) whereas smaller and larger adult dogs (n = 5 and n = 4, respectively, out of 11 adults) stem from the well house in Insula 8 (“Brunnenhaus”). The two dogs from Vindonissa (HAV1, HAV2) found in a burial context were small sized although HAV1 was a juvenile individual which likely had not attained its full size yet (see Fig. S2).

Four small adult dogs stand out morphologically. Dog HAL16/HAL10 from well MR 12 (“Sodbrunnen MR 12”) was abnormally short-legged (i.e. brachymelic, as confirmed by two bone elements). Further two dogs HAU12 and HAU14 from well house in Insula 8 (“Brunnenhaus”) were brachymelic too, but additionally the skull of subject HAU14 showed the characteristic traits of a brachycephalic (“short-headed”) dog (Table S1). To our best knowledge this is the first report of brachymelia and brachycephaly in dogs from this part of the Roman Empire (e.g. Roman Switzerland). Additionally, one small dog (individual HAU2) was slender. Altogether, four small adult dogs from a total of ten small dogs (seven adults) showed particular morphologies.

### Carbon and nitrogen isotope analysis

**Figure 2:**
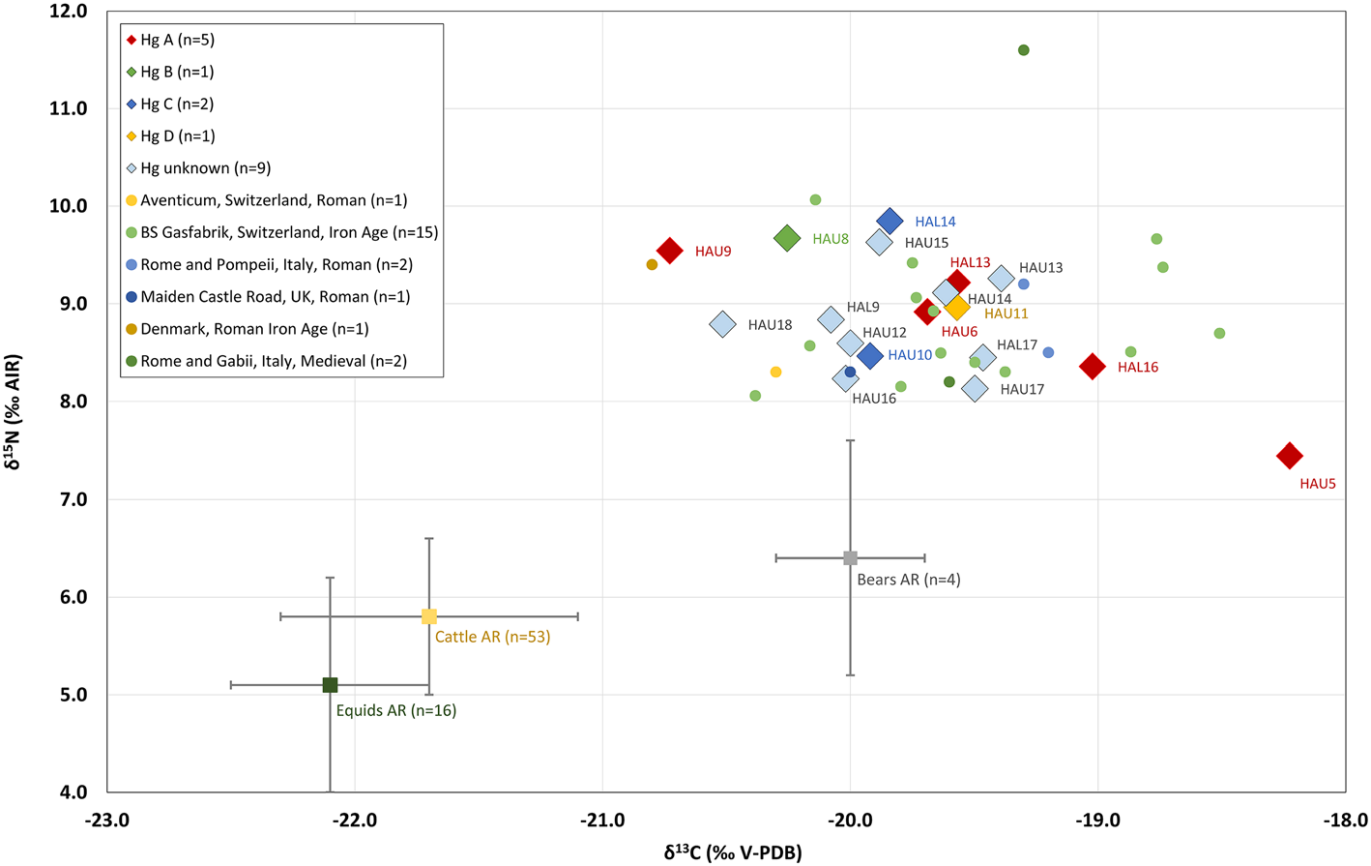
δ^13^C and δ^15^N values measured in bone collagen sampled from dog skeletal remains from Augusta Raurica (AR). Symbol shades denote haplogroups (Hg) (**A**–**D**) and unknown haplogroups. Isotopic values are plotted against herbivore and herbivorous omnivore stable isotope data from the same site (mean ± 1SD) and selected dog stable isotope data from Roman contexts across Europe and from the Late Iron Age site of Basel-Gasfabrik (2nd-1st century BCE), located < 15 km from Augusta Raurica.

Collagen of all 18 samples fulfilled the quality criteria for ancient collagen as suggested by Ambrose^65^ and van Klinken^66^. They yielded between 2.9 and 17.0% of collagen, 28.6 to 44.2% of C, 10.5 to 16.0% of N, and had atomic C/N ratios of between 3.1 and 3.4. The analytical data are listed in Table S1. The δ^13^C and δ^15^N values of the dog samples from Augusta Raurica varied between − 20.7 and − 18.2‰ and 7.4 and 9.8‰ respectively, and are in the range of published stable isotope data for dogs (see Fig. 2) indicative of an omnivorous diet^56,67–72^. The difference in δ^13^C and δ^15^N between dogs and herbivores from Augusta Raurica^73^ accounts for almost one trophic level^74^ indicating also substantial animal protein intake. δ^15^N variation was limited to 2.4‰, and δ^13^C showed a range of 2.5‰. Most samples clustered within a range of 1.7‰ in respect to both δ^13^C and δ^15^N, however, with the exception of outlier value HAU5 (Grubbs’s test (2-sided): δ^13^C: *Z* = 2.708, *p* = 0.038; δ^15^N: *Z* = -2.260, but *p* = 0.265). This large adult individual displayed a combination of low δ^15^N and high δ^13^C (Fig. 2) indicating a different dietary signature. Three dogs derive from features other than wells. Two of these are of earlier date (HAU9, HAU18) and displayed moderate but statistically significant differences to the dogs from wells (dating to the 2nd half of the third century CE) in respect to δ^13^C (Student’s t-test: *t*(15) = 2.698; *p* = 0.017) suggesting potential differences between these time periods.

### Mitochondrial DNA D-loop diversity

**Figure 3:**
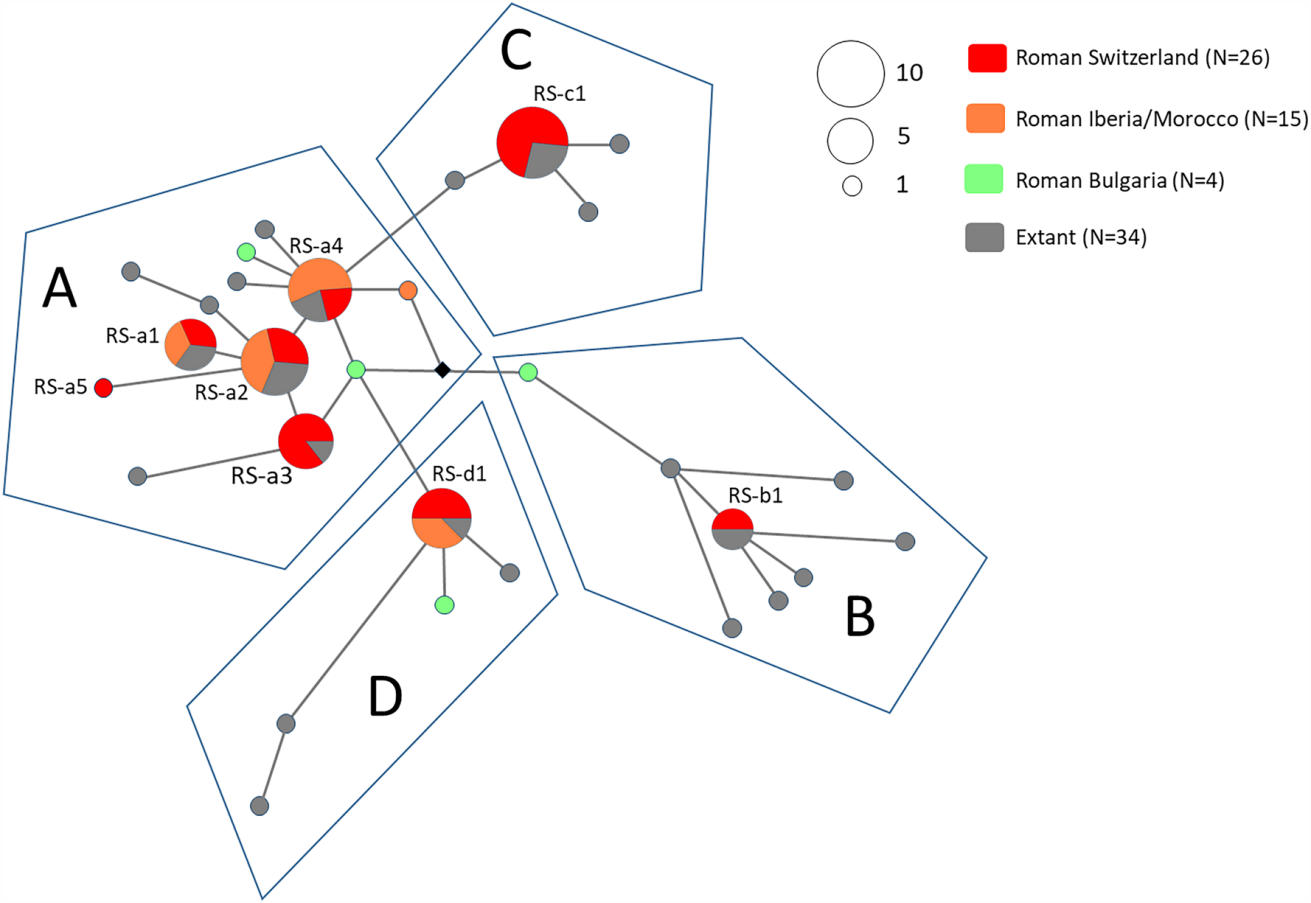
Median-Joining Network of Roman (n = 45) and modern (n = 34) mtDNA sequences (82 bp). Haplogroups (**A**–**D**) follow nomenclature of Duleba et al. (2015) based on whole mitochondrial genomes. Eight different haplotypes were found in Roman Switzerland. Median vector (filled rhombous) denotes hypothetical haplotype. Further details as listed in Table S2.

**Figure 4:**
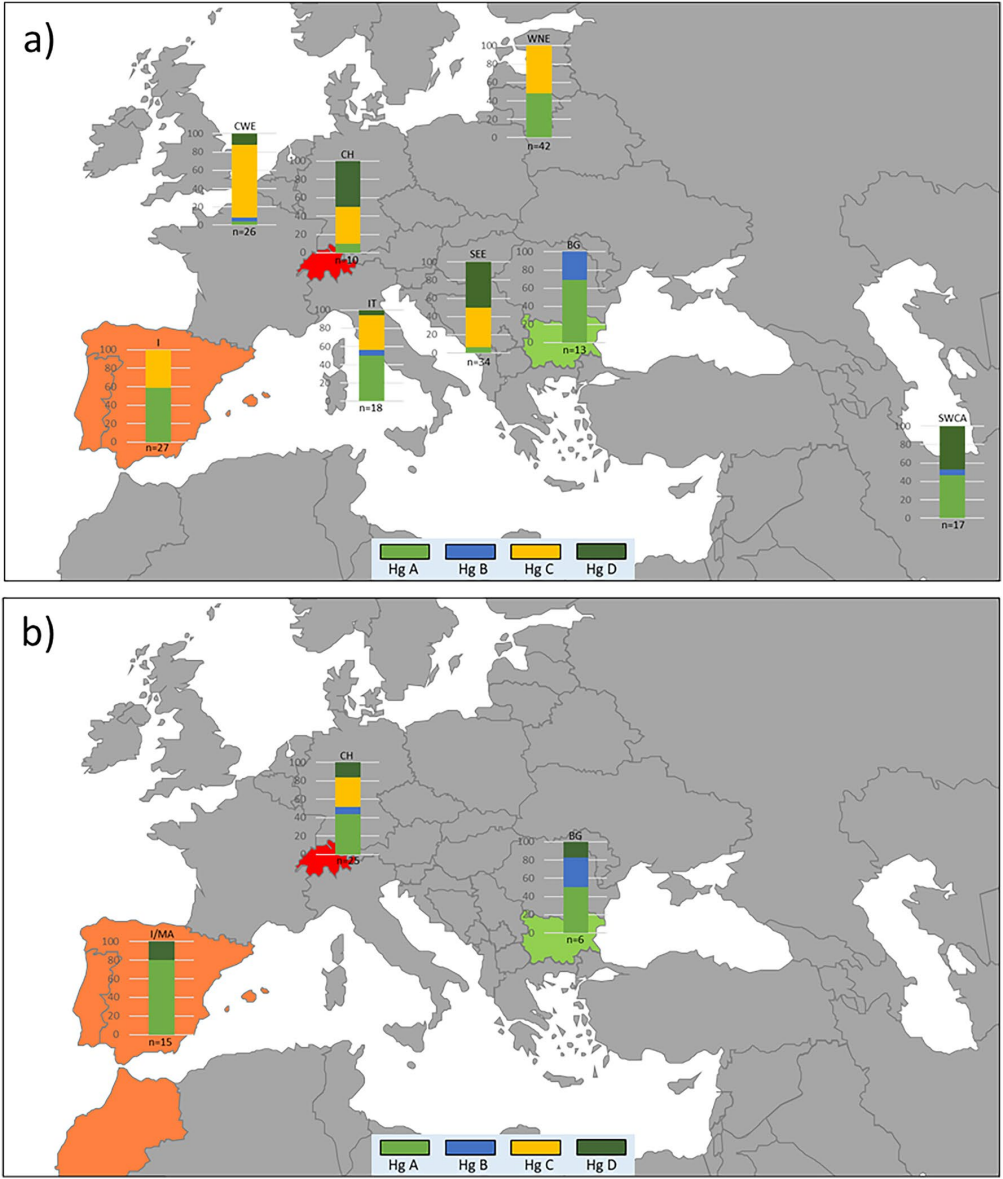
Visualization of haplogroup (Hg) (**A**–**D**) frequencies (0–100%, y-axis) in pre-Roman (a) and Roman (b) times in Eurasia: Iberia (I), Iberia/Morocco (I/MA), Switzerland (CH), Bulgaria (BG), Italy (IT), Central Western Europe (CWE), Southeast Europe (SEE), Western Northern Europe/Russia and Southwest and Central Asia (SWCA) as listed in Table S2. Free available map downloaded from slidelizard.com (https://slidelizard.com/en/blog/powerpoint-world-map) and edited using Microsoft Office 2016.

PCR amplification of the short mtDNA D-loop segment (97 bp, without primer) was successful in 24 dogs (out of 26) from Augusta Raurica and in one dog (out of two) from Vindonissa. Attempts to obtain a longer D-loop fragment (255 bp, without primer) failed in all samples positive for the shorter fragment. In total, 8 haplotypes could be assigned to one of the four major haplogroups A-D (Table 1, Tables S1, S2, Fig. 3, 4). Five haplotypes (RS-a1 – RS-a5) clustered within haplogroup A (11 dogs, 44%) which constitutes the most diverse dog haplogroup known today^36^. Haplotype RS-a5 (HAU7) is a private variant matching no identical sequence in public databases. Further, haplotype RS-a3 found in three dogs from Augusta Raurica (HAL2, HAL8, HAU3) is not shared with any other Roman dog (Fig. 3, Table S2). Three haplotypes clustered within haplogroup C (RS-c1, 8 dogs, 32%), D (RS-d1, 4 dogs, 16%,) and B (RS-b1, 2 dogs, 8%) (Fig. 3). The relatively high haplotype (0.86 ± 0.04) diversity is comparable to genetic diversity indices reported from modern native breeds from Portugal and Iran^75,76^.

### Haplotype distribution between Roman dogs and relatedness to pre-Roman dogs

To date, only two genetic studies of Roman dogs from Iberia/Morocco^20^ and Bulgaria^5^ are available. Consequently, dog mtDNA sequences from Augusta Raurica and Vindonissa were compared to mtDNA sequences from these two studies (Table S2, Fig. 3, Fig. S4). Two haplogroups were found in dogs of Roman Iberia/Morocco^20^ and, except for one (LYEP60), all their haplotypes are shared with dogs from Augusta Raurica and Vindonissa (e.g., RS-a1, RS-a2, RS-a4; and RS-d1) (Fig. 3). Four dog samples from Roman Bulgaria clustered within haplogroups A, B and D^5^ but haplotypes were neither shared with Augusta Raurica and Vindonissa nor with Iberia/Morocco.

**Figure 5:**
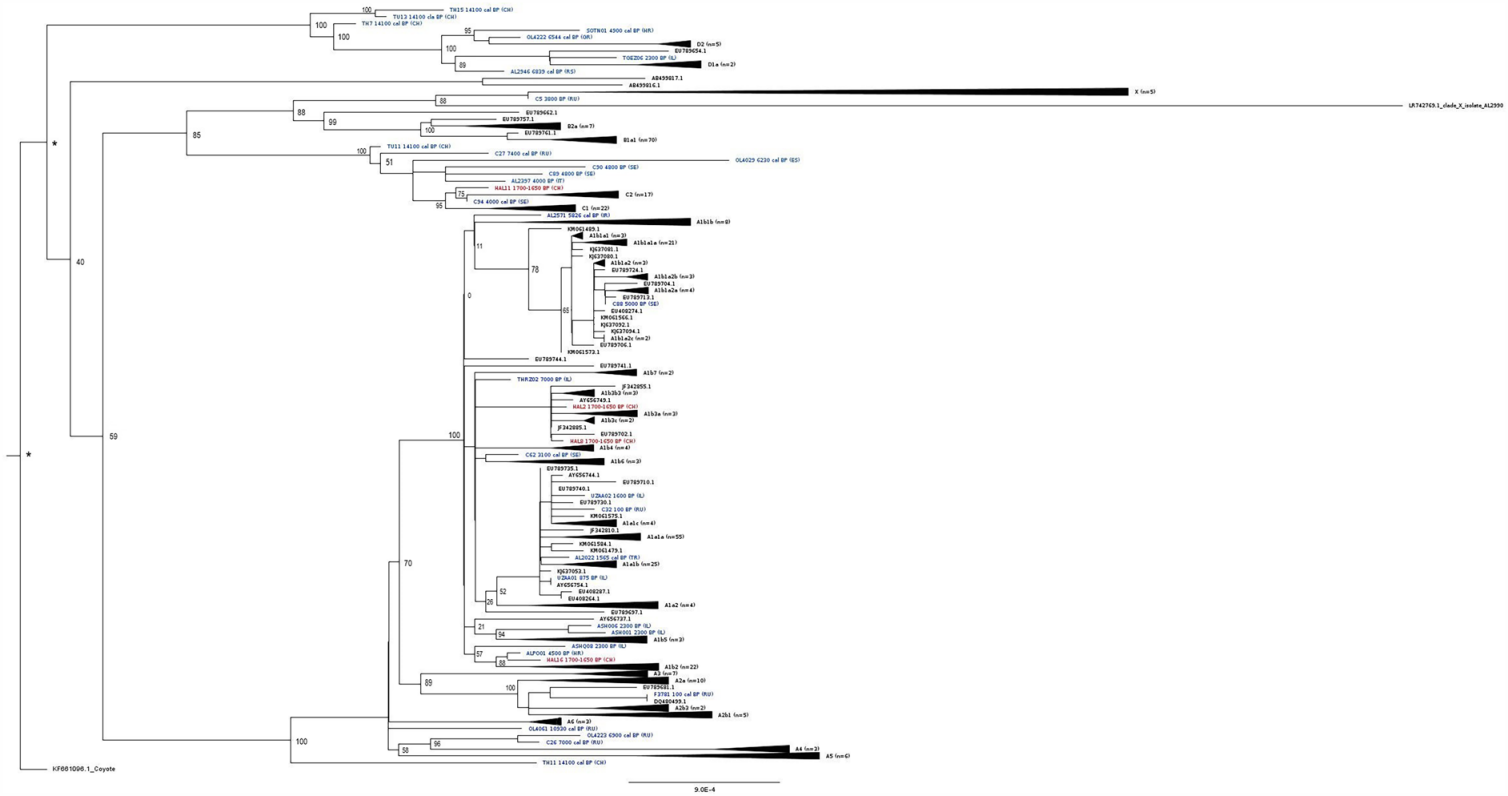
Maximum-likelihood tree of mitochondrial dog genome sequences. The tree contains four mitogenomes from Augusta Raurica (this work, in red text) along with published mitogenomes of modern (n = 512) and ancient (n = 38) dogs as listed in Table S6. An asterix (*) indicates no bootstrap values provided as the outgroup was used for tree rooting after inference. Bootstrap values (in percentage, from 500 replicates) are provided for internal branches of the tree.

By comparing Roman with pre-Roman sequences, shifts in haplotype frequencies were observed. For example, while most haplogroup A sequences affiliate with cluster “RS-a4” in pre-Roman Iberia/Morocco, a second cluster “RS-a2” appears with increased numbers of sequences and haplogroup D appears for the first time in Roman Iberia/Morocco (Table S2, Fig. S4). Similarly, haplogroup A and B haplotypes from Roman Bulgaria differ from pre-Roman haplotypes, and haplogroup D emerges for the first time in Roman Bulgaria (Fig. S4, Table S2). In Roman Switzerland new A and D haplotypes replace haplotypes from previous periods and haplogroup B is newly introduced (Fig. S4, Table S2). Interestingly, cluster “RS-d1” with shared sequences between Roman Iberia and Switzerland, is the most frequent D cluster in pre-Roman Southeast Europe (Table S2). While some haplogroup C lineages may have persisted through times in Switzerland (Fig. S4) others are new (see mitogenome HAL11 1700–1650 (CH), Fig. 5).

Shifts in the proportion of haplogroups are also observed (see Fig. 4): an increase in haplogroup A and D frequencies, the disappearance of C lineages in Roman Iberia/Morocco^20,38^; a decrease in haplogroup A and an increase in haplogroup D frequencies in Roman Bulgaria^5^. Notably, Bulgaria preserves the highest ratio of haplogroup B (ca. 30%) over time (Fig. 4). An increase in haplogroup A and B ratios, and a reduction of haplogroup D proportions is noted in Roman Switzerland. Pre-Roman frequencies of haplogroups A, B, C and D vary considerably between regions in Eurasia and contrast to frequencies reported from modern dogs (see Fig. S5).

### Relationships between phenotypes, genetics and diet

No obvious relationships between haplotypes, sizes, age or isotopic signatures was detected. Haplotype and accordingly haplogroup distribution did not show any particular association with size groups (Figs. S2 and S6). Stable isotope ratios of small, medium and large dogs overlapped with no apparent trend, that is, the dogs fed on similar, slightly varying omnivorous diets with small differences irrespective of their size (Fig. S3). Interestingly, stable isotope values of the three smallest dogs with abnormally short limbs HAL16/HAL10 and HAU12 (brachymelic) and HAU14 (brachymelic/brachycephalic) were within the same range and the only dog (HAU5) with outlier stable isotope values (Fig. 2, Table S1) showed no particular exceptional morphologies besides being large.

### Shotgun sequencing, pathogen screening and recovery of four mitogenomes

Shotgun sequences from 14 dog individuals from Augusta Raurica were generated. In general, the content of endogenous dog DNA was low (Table S5). The DNA displayed the damage pattern expected for aDNA (see Supplementary Methods). The genome-wide data were screened for pathogens (see Supplementary Information)^77^ but no bacterial or viral pathogens were detected. High-coverage mitochondrial genomes could be assembled for samples HAL2, HAL8, HAL11, HAL16. A maximum likelihood tree was constructed to view their position within other dog mitogenomes (Fig. 5). The tree contained published whole mitochondrial genome sequences from modern and ancient dogs representing the four well-supported major clades A, B, C and D^78^ and the new proposed clade X found in ancient Siberian dogs^79^ (see Table S6). One Roman mitogenome HAL11 located within clade C (close to C2), and three within clade A1: mitogenome HAL2 and HAL8 were slightly different from each other (otherwise identical based on the D-loop fragment) and clustered within sub-haplogroup A1b3 whereas mitogenome HAL16 clustered within sub-haplogroup A1b2 (see Fig. 5). PCR-based haplogroup classification based on the short D-loop fragment for individuals HAL2, HAL8, HAL11 and HAL16 was confirmed by their respective mitogenomes. Interestingly, the tree position of the four Roman mitogenomes differed from the two available Palaeolithic mitogenomes from Kesslerloch cave in Switzerland that cluster within C and A (TU11 and TH11, respectively)^78,80^.

## Discussion

The present study used an interdisciplinary research approach to analyze a large number of Roman dog individuals including dogs deposited in two wells that likely lived contemporaneously around the mid-third century CE in the town of Augusta Raurica, Germania Superior, a unique assemblage of bone deposits in Northern Switzerland. Osteometric analysis of all individuals (n = 33), revealed a broad range of sizes providing the first morphometric information from a large population of dogs living on the edge of the Roman Empire. The dogs were classified into three size categories, e.g. small (n = 10), medium (n = 17) and large (n = 6) and included young and adult individuals. Overall, the dogs show a similar size variation to dogs across other Roman regions and sites, e.g. Pompeii (Italia)^24^, Vindolanda (Britannia)^17,81^, and Hispania Tarraconensis^16,17,24^. The detection of three small-sized adult and abnormally short-limbed dogs (HAL16/HAL10, HAU12, HAU14) one of which with a brachycephalic skull (HAU14), and a slender dog (HAU2), denotes distinctive morphotypes of small dogs co-occurred at Augusta Raurica. A similar short-limbed small brachycephalic dog was reported from the Roman cemetery of Llanos del Pretorio (Córdoba, Spain)^23^, implying that such combined traits were not unique and restricted to one particular region. This raises the question of where this dog type was first bred and whether they were indeed lapdogs, as previously suggested for these kinds of small individuals^23,24,82^. Two dogs, one juvenile (HAV1) and one adult (HAV2) from the military camp of Vindonissa were small and shared no particular phenotype but were found in burial contexts, potentially being esteemed household dogs of legionary people.

In contrast, it is rather surprising to find a brachycephalic dog with such a particular trait disposed in a well. Was this dog not considered as something special (e.g. prestige pet?) and worth a proper burial? Was it subject to a ritual act, or was it a precipitous disposal with another background? The same questions may also apply to the slender dog. The remains of several other wild and domestic animals (including horses, pigs and bears) were also disposed of in the wells along with the dogs^44,47^, raising questions around the way that these deposits should be interpreted. Various theories are being discussed for this phenomenon such as “only” waste disposal, victims of war or epidemic, political crisis, the contamination of water or ritual deposits^44,48^. The short period of time during which the animals were deposited and the relatively large number of dogs and other animals disposed in the wells suggest that an epizootic disease was a potential cause. Shotgun DNA sequence analysis of dog viral and DNA based pathogens was, however, negative. The general low quality and quantity of endogenous nuclear DNA of the samples might explain the negative screening results for viral and bacterial pathogens. Or, pathogens attacking primarily soft tissues remained unperceived. Therefore, we cannot completely rule out infectious disease or other pathologies as cause of dog death and disposal.

The capacity in dogs to digest starches has been demonstrated since the transition to the Neolithic^6,83^ suggesting that dogs followed similar diets as humans with substantial proportions of vegetal food components besides animal proteins. It is known that the Romans were feeding their dogs differently according to a number of factors, including function and age, but most likely the majority of Roman dogs were relying primarily on table scraps^31^. Stable carbon and nitrogen isotope ratios obtained from dogs of Augusta Raurica of mixed size, age and haplotype composition (Fig. 2, Table S1) show that they fed on similar, only slightly varying diets. The data are consistent with a mainly mixed omnivorous diet including variable meat and vegetable proportions, similar to other Roman sites across Europe^23,67–72,84^ and the Celtic dogs from Basel Gasfabrik (150– 80 BCE), a site located close by Augusta Raurica (Fig. 2)^56^. This matches numerous archaeological, epigraphic and literary evidence for dog food in the Roman Empire, that show a large proportion of vegetal components, e.g. cereals, bread (made of barley, wheat or spelt), and a minor component of meat, bones, milk and whey (Harvey^31^). The substantial difference in δ^13^C and δ^15^N values between herbivores from Augusta Raurica, i.e. horses^73^ suggests that the proportion of animal proteins in the dogs’ diet should not be underestimated. The low isotopic variability may support the hypothesis that size, age or function, may have only moderately impacted stable isotope compositions at Augusta Raurica. However, stable isotope compositions as found in the dogs from Augusta Raurica do not allow for reliable assignments of functions to individual dogs. The isotopic signals show no significant differences between the small dogs with particular morphologies (brachymely and brachycephaly) and the others (Fig. 2). This could indicate that these particular dogs did not receive any special food. Two dogs (HAU9, HAU18) with earlier dates (ca. 200 CE and 70–100 CE, respectively) and different archaeological contexts displayed more negative δ^13^C values compared to the dogs disposed of in wells. Sample sizes are very small, one tentative explanation could be that the two dogs fed on specific (plant) components low in δ^13^C that eventually became scarce by the 2nd half of the third century CE when the town of Augusta Raurica supposedly was in decline, another explanation could be diachronically varying climatic, e.g. more humid, conditions. The only distinct outlier, HAU5, fed on an exceptionally low-protein and more plant based diet (Fig. 2). One possible explanation would be a diet including C_4_ plants such as millet, reported to be grown at Augusta Raurica^39^. The combination of a low meat and high plant intake could be seen as an indication for unbalanced nutrition in the individual HAU5, but the reasons behind this remain unclear since there are no unusual findings in archaeology, morphology or aDNA.

Mitochondrial DNA variation in a 97 bp fragment of the control region in 25 individuals points to a genetically diverse dog population at Augusta Raurica and Vindonissa. Eight haplotypes representing all four widely recognized major haplogroups A-D^34^ were detected but no association between haplotype and size/morphology was apparent. The concomitant presence of all the four major haplogroups in this dog population is remarkable as modern dog breeds generally do not harbor all four haplogroups at once^36,75,76,85^. The observed haplotype diversity corresponds to the increasing morphotype diversity in the Roman world^23^ and provides insights into a diversifying dog population in situ, with a sharp increase in effective population size starting around 2,500 years ago through the Roman era into modern times^36,78^. Therefore, we may assume Roman people likely propagated and dispersed dog lineages across the Empire into the Colonies (and back) as reflected in dog lineage diversity in Augusta Raurica and Vindonissa.

Some of the lineages from Iberia, Bulgaria and from Augusta Raurica were unique to different Roman sites and either went extinct, were subject to genetic drift or were not yet detected due to relatively small datasets especially for archaeological dogs. Because of the lack of genetic data from Roman Italy and also because of the rarity of mtDNA data from archaeological dogs elsewhere and from other time periods to compare with, interpretations are limited. However, some scenarios might be considered taking into account the known trade/contact routes in Roman Europe: the dogs from Augusta Raurica share many haplotypes with dogs from Roman Iberia/Morocco (haplogroups A and D), but not with Roman Bulgaria suggesting a connection between dog lineages from both regions. From the Median-Joining Network analysis of pre-Roman dog lineages (Fig. S4, Table S2) other links are conceivable such as A haplotype cluster “RS-a2” or cluster “RS-a4, might have reached Augusta Raurica via Italy. Interestingly, Chalcolithic to Bronze Age Italy shows frequencies of haplogroup A (50%) and C (42%) comparable to Roman Switzerland^86^. Pre-Roman and Roman Bulgaria is a known centre of haplogroup B^5^ which is also reflected in the haplogroup frequency distribution in Europe (Fig. 4). Comparison to some pre-Roman data from Southeast Europe in particular haplogroup B and D haplotypes are found at Augusta Raurica, hints to a connection also to that region. (Fig. S4, Table S2). The situation for haplogroup C lineages suggests potential continuity for some C haplotypes in Switzerland from the Neolithic (ca. 5500 – 2200 BCE) into Roman times (ca. 1st century BCE–4th century CE). Future research is needed to test these hypotheses.

We assembled four new mitogenomes from dogs of Augusta Raurica with three diverse A haplotypes and one C haplotype. This allows the subdivision of individuals in haplogroup A into sub-haplogroups A1b2 and A1b3 and in haplogroup C into sub-haplogroup C2 (assignment after Duleba et al.^36^). The four Roman mitogenomes differed from dog mitogenomes from Palaeolithic Switzerland (Fig. 5)^78,80^. The past history of dogs in Switzerland is marked by continuity and replacements of lineages but remains largely unknown/unresolved due to a lack of studies. The dogs of Augusta Raurica and Vindonissa fill, therefore, a small part of this knowledge gap.

The mtDNA diversity documented here in Roman dogs of northern Switzerland is only known from modern dogs. The high diversity of haplogroups and haplotypes concentrated at Augusta Raurica suggests that diverse maternal lineages were kept at the same time and place simultaneously. This is surprising as much of the diversity of today’s dogs is generally seen as the result of breeding activities over the last 200 years and is not expected to originate from earlier, e.g. Roman times, ca. 2000 years ago. Some dogs likely accompanied their owners to Augusta Raurica and Vindonissa (e.g. traders, legionary families, i.e. people known to come in from any part of the Roman Empire), underpinning the region as a trade and transport hub north of the Alps under the rule of Rome.

## Conclusion

This study has provided the first archaeozoological and biomolecular insights into a hitherto unstudied dog population from Augusta Raurica and Vindonissa, Germania Superior, emphasizing the utility of wells and burials as sources for the reconstruction of dog diversity in Roman times in general. Based on our data no function was assigned to the dogs. However, it is a reasonable assumption that dogs from wells were a mixture of free-roaming individuals and animals cared for by people. The dogs were highly variable in size and include dogs with short-limbs, slenderness and one extreme case of brachycephaly.

Overall, the dogs of Augusta Raurica were similarly omnivorous with a tendency towards animal proteins. Whatever the living conditions were, the dogs showed no sign of undernourishment (except maybe for outlier HAU5 with a lower animal protein ratio in its diet) in line with general observations from other Roman regions^30^.

Mitochondrial D-loop and mitogenome diversity suggests that diverse maternal lines were kept in Augusta Raurica and Vindonissa and probably had different geographical origins and haplogroup frequencies in the Roman period, showing a specific pattern compared to other European regions. Some of the dogs shared the same maternal lineage and, since they were living almost contemporaneously and all very likely in Augusta Raurica, they might have been closely related, raising questions of kinship and potential local breeding without excluding the possibility of mixing by chance.

### Supplementary Information

Supplementary Information 1.

Supplementary Information 2.

Supplementary Information 3.

Supplementary Information 4.

## Data Availability

Ancient partial mitochondrial DNA and whole mitochondrial genome dog sequences generated in the current study are publicly available from GenBank accession numbers OP082250-OP082275 and from the European Nucleotide Archive (ENA) with project number PRJEB55253, respectively. All raw data are available upon request.
