# Supplementary material for: A melting pot of Roman dogs north of the Alps with high phenotypic and genetic diversity and similar diets
Source: Sci Rep. 2023 Oct 13;13:17389. doi: 10.1038/s41598-023-44060-3 (PMC10575936; doi:10.1038/s41598-023-44060-3)
Supplement: Supplementary file 1 — Supplementary Information 1. [file 41598_2023_44060_MOESM1_ESM.docx]

**Supplementary Information for:**

**A melting pot of Roman dogs north of the Alps with high phenotypic and genetic diversity and similar diets**

Authorship

José Granado^1,3^, Julian Susat^2,3^, Claudia Gerling^1^, Monika Schernig-Mráz^1^, Angela Schlumbaum^1^, Sabine Deschler-Erb^1,4^, Ben Krause-Kyora^2,4^

^1^Department Environmental Science, Integrative Prehistory and Archaeological Science (IPAS), University of Basel, Spalenring 145, 4055 Basel, Switzerland.

^2^Institute of Clinical Molecular Biology (IKMB), Christian-Albrechts-University Kiel, Rosalind-Franklin-Strasse 12, 24105 Kiel, Germany

^3^These authors contributed equally: José Granado and Julian Susat

^4^These authors jointly supervised this work: Sabine Deschler-Erb and Ben Krause-Kyora

**Archaeological sites and samples**

The sites are located in the northwestern part of modern Switzerland (Fig. 1) which was part of the Roman Empire from the end of the first century BCE. The river Rhine formed the border of the Empire until 101 CE, then later the border was moved further north into southern Germany and the area became hinterland.

**Augusta Raurica**

Augusta Raurica (lat 47.533, long 7.7167; today Augst/Kaiseraugst) was founded as a colonial town in the late first century BCE^1^. During its heyday in the second and the first half of the third century CE its population may have reached 9000–15,000 inhabitants^2^ consisting of “natives” and people from across the Empire. We know that widely travelled traders, artisans and public officials came to Augusta Raurica for business reasons^1^.

To date more than 480’000 animal bone fragments from Augusta Raurica have been analysed^3^. Several dogs were discovered in two defunct wells, together with other wild and domestic species, and a few dogs stem from other structures (Table S1). A minimum number of 42 individual dog skeletons were found in well MR 12 (“Sodbrunnen MR 12”) (Fig. S1) from the Lower Town of Augusta Raurica which was a centre of production with workshops, manufacturing and handicraft. Dogs represent about 56% of the domestic fauna in the well (NISP n=5827). Another 53 sixteen dog skeletons originate from a well house in Insula 8 (“Brunnenhaus”) (Fig. S1) located in the Upper Town; an area considered to be the centre of higher society and the upper-classes. Dogs from this well represent about 40% of the domestic fauna (NISP n=11936). The fillings of these two wells can be dated to the second half of the third century CE and suggest that the accumulation of the specific finds took place within a relatively short period of time^4,5^. This is important to note, because other archaeological evidence suggests the city of Augusta Raurica was declining in the second half of the third century CE. One dog (HAL17) from the Lower Town was obtained from a pit (“pit G14”, "Überbauung Schürmatt 2. Etappe, Friedhofstrasse") dating to the late third to the first half of the fifth century CE^6^. Another dog sample (HAU9) was obtained from a hypocaust room within a peristyle house in Insula 27 in the Upper Town, dating to ca. 200 CE^7^, and a further individual (HAU18) was found in a pit (“pit 3 and 5”; Phase B4, Insula 53) dating to 70-100 CE^8^.

**Vindonissa**

Vindonissa was the only legionary camp (founding year 14/15 CE) that was located in Switzerland (lat. 47.476728, long. 8.213606, today Windisch). About 6000 soldiers from different regions of the Roman Empire and about 3000 civilians were living there. In 101 CE, the camp was abandoned and the legion was moved to the north to the Obergermanisch-Raetische limes^9^. Up to 35000 bone fragments from Vindonissa have been examined so far^10-12^. Two small dog skeletons were recovered from special find contexts at Vindonissa (Table S1). The first (HAV1) was recovered from a rectangular pit within a Roman cemetery from “Brugg-Remigersteig” without grave goods and dates from the second third of the first century CE^13^. The other (HAV2) was a headless dog deposited in an officer’s house along the south west wall of the main road via Pretoria. Half a meter away in line with the dog skeleton, the skull of a sheep/goat was found, and 1.5-2 m away two separate neonate burials were recovered. The neonates and the dog with the sheep/goat skull stratigraphically belong together^14^. The dog deposition can be dated to the last third of first century CE^14^. The burial contexts of these two dog skeletons point to a particularly close relationship between these individuals and their keepers.

All the dates are based on archaeological context and co-finds. Archaeological background information of the dog samples are provided by different authors^4-8,14^. Details of the dog samples, including archaeological, metrical, genetic and stable isotope data are summarized in Table S1.

**Identification of bones**

Dog remains were determined and assigned to skeletal element based on a comparative collection established at the Integrative Prehistory and Archaeological Science, University of Basel (IPAS) and recorded in the Ossobook database^15^. Most of the individuals in this study were disposed in two wells as whole or partial carcasses, however their individual context was disturbed after the disintegration of soft tissues and sedimentation, so that it was sometimes impossible to assign all bones, in particular small compact bones such as hand and foot bones or ribs to single individuals with a certainty. It was also not easy to reconnect cranial and postcranial skeleton fragments/elements for the dogs that were very similar in size and age. Therefore, in this study only bones from clearly identified individuals have been chosen for metrics, aDNA and stable isotope analysis, e.g. fifteen dog individuals from well MR 12 (HAL1-HAL9, HAL11-HAL15, HAL10/HAL16) and sixteen individuals from well house in Insula 8 (HAU1-HAU8, HAU10-HAU17) (Table 1, Table S1).

**Measurements/metrics**

The wither's height was calculated by greatest length (GL) of long bones that were preserved in one piece according to the methods of Clark^16^ and Koudelka^17^. Due to fragmentation of many bones it was unavoidable to use different bone elements for calculations (see Table S1). Dogs were classified into the size groups: small (<39 cm), medium (40-59 cm), or large (>60 cm) based on the American Kennel Club (AKC)^18^ which is based mainly on modern breeds. All measurements on bones were taken with electronic calipers. Size distributions are visualized in Fig. S2 and also plotted against stable carbon and nitrogen isotope ratios (Fig. S3).

**Assessment of skeletal abnormalities**

Brachymelia, also called achondroplasia, a short-limbed dwarfism, is a genetic disorder associated with a mutation consisting of a duplicate copy (retrogene) of the gene encoding fibroblast grow factor 4 (fgf4)^19,20^. Characteristics include abnormalities of cartilage and/or bone growth and development with early calcifying of growth plates producing shortened legs typical for recent breeds like Dachshund, Pekingese, Basset hound, Corgi etc. The shortening of the long bones, especially by zygopodium, can be assessed by macroscopical examination. Examples of achondroplasia in dogs were documented at Roman urban sites in Europe e.g.^21^.

Two samples from the Lower Town of Augusta Raurica, one right humerus (HAL10) and one left (HAL16) were abnormally short. Both elements belong to the same individual (HAL16/HAL10) and, as expected, they share the same haplotype. Two further individuals from the Upper Town (HAU12, HAU14) were also short-limbed. Inspection of skull elements of the three short-limbed individuals revealed a brachycephalic phenotype only for individual HAU14 (Table S1). One further dog (individual HAU2) showed slender bones (bones were very narrow in breadth in comparison to length compared to the other dogs of the study (Table S1)). Limb bones from all other dogs showed no abnormalities or particular features. Most skull bones and teeth are under investigation in more detail elsewhere.

**Assessment of age (juvenile, adult)**

Tooth wear and replacement and epiphyseal fusion were used for age determination^22, 23^. For adult dogs precise age determination can be achieved by counting of incremental lines in cementum of dog’s teeth^24,25^, this method was used additionally only for few individuals. For this study the following age groups were used: juvenile/young dogs < 24 months and adult dogs >24 months.

**Stable isotope analysis**

Sample preparation and collagen extraction was conducted on 18 dog bone samples at IPAS. Bone samples were cut and mechanically cleaned using a dental drill. Collagen extraction followed the methodology outlined in^26-28^ with exclusion of the ultrafiltration step. Ca. 500 mg of sample material was demineralized in 10 ml of 0.5M HCl at 4 °C for two weeks. Samples were rinsed with ultrapure water before treatment with 10 ml of 0.1M NaOH at 4 °C for about 24 h. After rinsing to reach neutral conditions, 4 ml of ultrapure water and 200 μl of 0.5M HCl were added and samples gelatinized at 70 °C for 48 h. The solution containing the dissolved collagen was filtered using Ezee Filter separators (Elkay, UK) with pore size 60-90 μm, frozen at -20 °C, and lyophilized for 48 h. Between 0.7 and 0.8 mg of freeze-dried sample were weighed into tin capsules, and their carbon and nitrogen isotopic compositions (δ^13^C, δ^15^N) were determined by Elemental Analysis – Isotope Ratio Mass Spectrometry (EA-IRMS) using an INTEGRA2 instrument (Sercon Ltd., Crewe, UK) at the Department of Environmental Sciences, University of Basel. All samples were analysed in duplicates. Carbon and nitrogen isotope data were blank-, linearity, and drift-corrected and normalized to the VPDB and AIR scales respectively. Reproducibility of internal and external standards was better than ±0.1 ‰ for δ^13^C and ±0.25 ‰ for δ^15^N. The obtained isotope data were compared to published isotope values derived from dogs of Late Iron Age Switzerland^29^ and across Roman Europe^30-35^ (Fig. 2).

**DNA work and analysis at Basel:**

Workflow was strictly unidirectional, starting with pre-PCR work (sample preparation, DNA extraction/purification and PCR setup) performed in dedicated, physically separated laboratories followed by post-PCR treatment in a different building according to ancient DNA standards. Surfaces and tools were cleaned with bleach and UV irradiated. Disposable plastic ware was UV-irradiated before use. A minimum of two independent DNA extracts per sample were generated. Negative PCR controls were included and at least two PCR products per extract were sequenced to validate each target.

**Sample preparation and DNA extraction**

A subset of dog samples from the Lower Town (16 samples, =15 dogs) and Upper Town (11 samples, =11 dogs) of Augusta Raurica and two samples (=two dogs) from Vindonissa were chosen for mtDNA analysis. Bone powder was obtained by drilling with a Dremel® tool at moderate speed after removal of a small area of the outer surface (Table S1). DNA was extracted using a silica-based protocol followed by ultrapurification with 30 kD filter units as described elsewhere^36^. At least two independent DNA extractions per sample were performed and for every four samples one extraction control/blank (no bone powder) was included. Mean yield of DNA extract was about 150 µl per sample.

**PCR conditions and sequencing**

Primers ”Canis F2” 5’TGGTTTGYCCCATGCATA3’ and ”Canis R4” 5’TGATTAAGCCCTTATTGGA3’^37^ were used to amplify a short fragment of 134 bp (i.e. 97 bp, primers excluded) between positions 15554-15687 of mitochondrial D-loop region (HVRI) of the dog reference mitochondrial genome NCBI accession number NC_002008.4^38^. The amplicon harbours sufficient polymorphic information to support haplogroup identification^37^. PCRs were performed in 25-µl reactions containing 2 μM of each primer, 1× GeneAmp 10× PCR Gold Buffer (150 mM Tris-HCl, 500 mM KCl, pH 8.0), 2 mM MgCl2 (all Applied Biosystems, Hombrechtikon, Switzerland), 400 μM dNTP Mix (Promega, Dübendorf, Switzerland), 4.4 μg/ul BSA (bovine serum albumin, Roche, Basel, Switzerland), 8-10 μl DNA extract and 1.5 U of AmpliTaq Gold. After an initial denaturation step for 12 min at 94 °C 70 PCR cycles were run at 94 °C for 1 min, 53 °C to 54 °C for 1 min, 72°C for 1 min, followed by final extension step at 72 °C for 5 min. In a set of PCR runs one extraction blank (instead of DNA template) was included for every forth sample. For every extraction at least two PCR products were generated. PCR amplification of longer D-loop fragment (298 bp, positions 15410-15707, i.e. 255 bp without primer) was attempted by using primers L15426int 5’CTCCACCATCAGCACCC3’ and H15692int 5’GATGGTTTCTCGAGGCA3’^39^ under the same PCR conditions as for the shorter fragment. PCR products were separated by agarose gel electrophoresis and amplicons of the expected size were gel-purified and sequenced as previously described^36^.

**MtDNA sequence analysis; haplogroup/haplotype assignments**

Sequences were edited by eye and aligned to the reference mitochondrial dog genome (NC_002008.4) with BioEdit and majority-rule consensus sequences were built. Summary statistics were performed with Arlequin 10.0^40^. The unique positive sample from Vindonissa (HAV2) and sample HAU9 (fom Insula 27, Upper Town) were excluded from analysis because they both were from an earlier time period than the other samples (see Table S1).

Sequences were assigned to distinct haplotypes based on polymorphic sites that were assessed from sequence alignments to the mitochondrial dog reference sequence NC_002008.4. Nomenclature of haplogroups followed Duleba et al. 2015^41^ based on diagnostic polymorphic sites within D-loop region (Table S1). Haplotype and haplogroup distribution were compared to size distribution (Fig. S2 and Fig. S6). To study relationship among haplotypes, two Median-Joining Networks (MJN) were constructed with NETWORK version 5.0.0.3^42^. Nucleotide substitutions were weighted 10 for transitions and 30 for transversions, respectively (see Pires et al. 2018^43^).

In the first MJN analysis, published sequences from Roman Iberia/Morocco (n=15)^43^ and Bulgaria (n=4)^44^ together with selected modern dog sequences (n=34)^41,45-53^ with defined haplogroup assignment as reference were compared with the Roman dog sequences from Augusta Raurica/Vindonissa (n=25). Sequences were trimmed to the same length and overlapped by 82 bp (Fig. 3) (Table S2).

For broader historical context a second MJN analysis was performed: Roman dog sequences from this work (n=25), Iberia/Morocco (n=15) and Bulgaria (n=4) (as above mentioned) were compared with a broader panel of published sequences, including pre-roman samples from Italy (n=18)^39,54-56^ and from Switzerland (n=8)^57-58^, Iberia (n=26)^60^, Bulgaria (n=12) ^44^,Central Western Europe (n=25)^37,58,59,61,62^, Southeast Europe (n=34)^37,59,63^, Southwest and Central Asia (n=17)^59,63^, Western Northern Europe/Russia (n=38)^58,59,63-65^ and reference sequences from defined modern dog lineages (n=34) (as above mentioned) (see Table S2). As a consequence of including the pre-roman Italian dogs into the MJN analysis sequences overlapped by only 44 bp after trimming but still retained sufficient diagnostic positions/polymorphic sites for reliable canine haplogroup assignments (Fig. S4).

Time related haplogroup frequencies were calculated for Roman and pre-Roman dogs based on published sequences including sequences from this work (Table S2) (see Fig. 4). Haplogroup frequencies were estimated for modern dogs (Fig. S5) based on published sequence numbers: Europe (n=206) Hg A (n=140), Hg B (n=36), Hg C (n=12) Hg D (n=18); Southwest Asia (n=90) Hg A (n=51), Hg B (n=32), Hg C (n=5) and Hg D (n=2) (compiled in Savolainen et al. 2002^47^). Further: Europe (n=77) Hg A (n=50), Hg B (n=18), Hg C (n=9) and Hg D (n=0); Southwest Asia (n=340) Hg A (n=230), Hg B (n=80), Hg C (n= 24) and Hg D (n=6) (compiled in Frantz et al. 2016^37^).

**DNA work and genetic analysis at Kiel:**

**DNA isolation, sequencing and postprocessing**

All lab work was carried out in a dedicated facility according to ancient DNA standards. Extracted DNA from 14 femur and humerus samples was sequenced (Illumina HiSeq4000 (2 × 75 bp)) and subsequently clipping and merging was performed as described in Krause-Kyora et al. 2018^66^. Reads that were shorter than 25 bp were discarded after merging. Endogenous DNA content was calculated based on mapping against the complete CanFam3.1 reference genome (GCF_000002285.3) (Table S3).

**Pathogen Screening**

The pathogen screening was carried out using MALT with the parameters described in Susat et al. 2020^67^. All samples were screened separately for the presence of viral and bacterial pathogens using custom databases containing complete genomes downloaded from NCBI. Hits against either one of these databases were inspected visually and no true positive pathogen sequences were identified during the screening process.

**Alignment against the *Canis lupus* mitochondrial genome**

All 14 samples were mapped against the *Canis lupus familiaris* mitochondrial genome (NC_002008.4) using BWA with the following command (Table S4)^68^:

bwa aln -n 0.01 -l 300 $INDEX $FASTQCM $OUT

where $INDEX is the reference, $FASTQCM is the input file and $OUT is the output file. Minimum mapping quality was set to 0. After mapping bam files were merged and duplicates were removed using DeDup with default parameters^69^. Subsequently damage patterns for the four best samples were generated using MapDamage^70^.

**VCF and FASTA generation**

VFC files displaying all genomic positions were generated for the four samples that exhibited sufficient coverage (HAL2, HAL8, HAL11, HAL16, Table S5) using GATK with the following command line^71^:

GenomeAnalysisTK.jar-T UnifiedGenotyper -R $REF -I $IN -out_mode EMIT_ALL_SITES -o $OUT

where $REF is the reference (NC_002008.4), $IN is the input BAM file and $OUT is the VCF output. The resulting VCFs were converted to FASTA using an in-house script with the following thresholds for the validation of each nucleotide position. Minimum coverage depth of 3x, minimum calling quality of 30 and minimum nucleotide frequency of 90% for a majority call. Only if all three parameters are met the respective nucleotide will be written in the FASTA file. Otherwise an N will be written.

**Multiple alignment using MAFFT**

The four genomes (HAL2 1700-1650 (CH), HAL8 1700-1650 (CH), HAL11 1700-1650 (CH), HAL16 1700-1650 (CH)) showed damage patterns typical for aDNA (Fig. S7) and were combined with 512 modern^45,46,49,58,72-77^ and 38 ancient^57,63,78^ references (Table S6) in one fasta file and MAFFT was executed with default parameters^79^.

mafft $IN > $OUT

Where $IN is the multifasta file and $OUT the output file containing the alignment. Subsequently GBLOCKS was used with default parameters to remove uninformative positions from the multiple sequence alignment^80^. After GBLOCKS redundant sequences were removed which resulted in 415 sequences for the identification of haplogroups/sub-haplogroups by maximum-likelihood analysis.

**Generation of a maximum-likelihood tree using RAXML**

A maximum-likelihood tree was generate using RAXML and the following command line^81^:

raxmlHPC -f a -x 12345 -p 12345 -#500 -m GTRGAMMA -s $IN -n $NAME -o $OUTGROUP

Where $IN is the output file from UCLUST, $NAME is the name given to the resulting tree files and $OUTGTOUP is the mitochondrial genome that was used as an outgroup for tree rooting (KF661096.1 Coyote). The resulting tree was visualized in FigTree^82^. For better visibility the branch of the outgroup was shortened manually (see Fig. 5). Nomenclature of haplogroup/sub-haplogroup follows Duleba et al. 2015^41^.


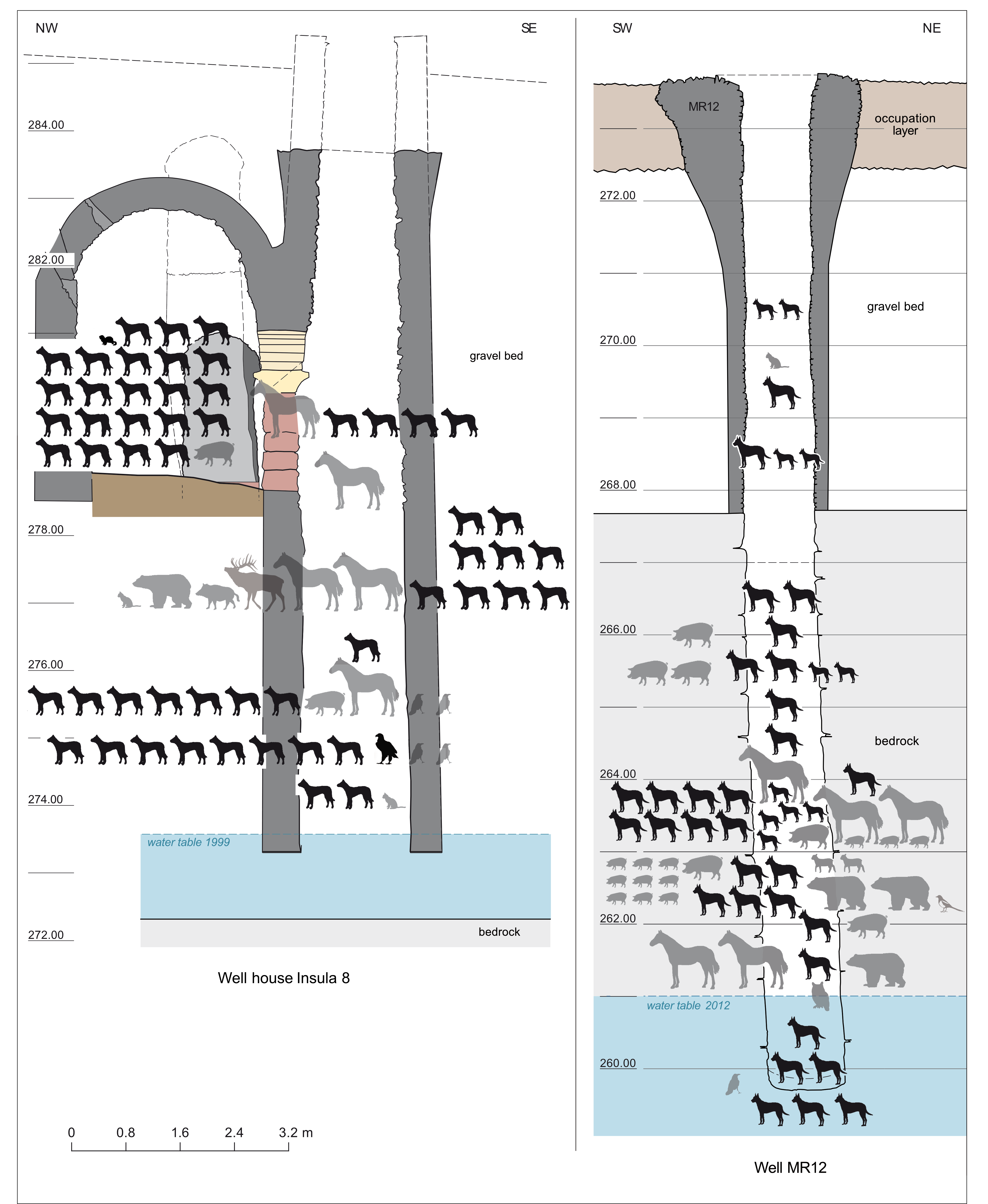


Figure S1. Subterranean well house in Insula 8 and well MR12. Dogs are coloured in black. The animal silhouettes represent the minimum number of individuals (MNI), mainly agreeing with complete or almost complete carcasses (modified from Gerling 2023^83^). Credit: image, Claudia Zipfel, Römerstadt Augusta Raurica.


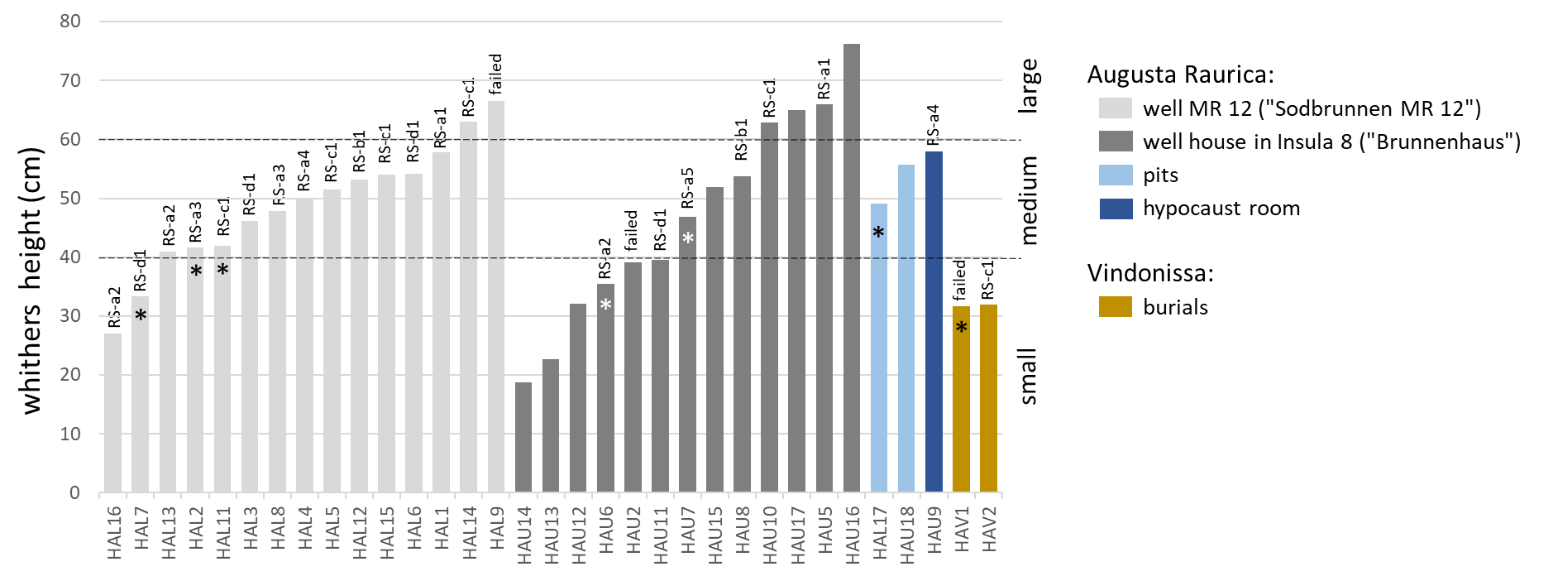


Figure S2. Size distribution of 33 dogs from Augusta Raurica and Vindonissa with 7 juvenile (*) and 26 adult animals. Haplotypes (n=22) are marked over the bars. Dashed horizontal lines denote size ranges. Dogs ID as listed in Table S1.


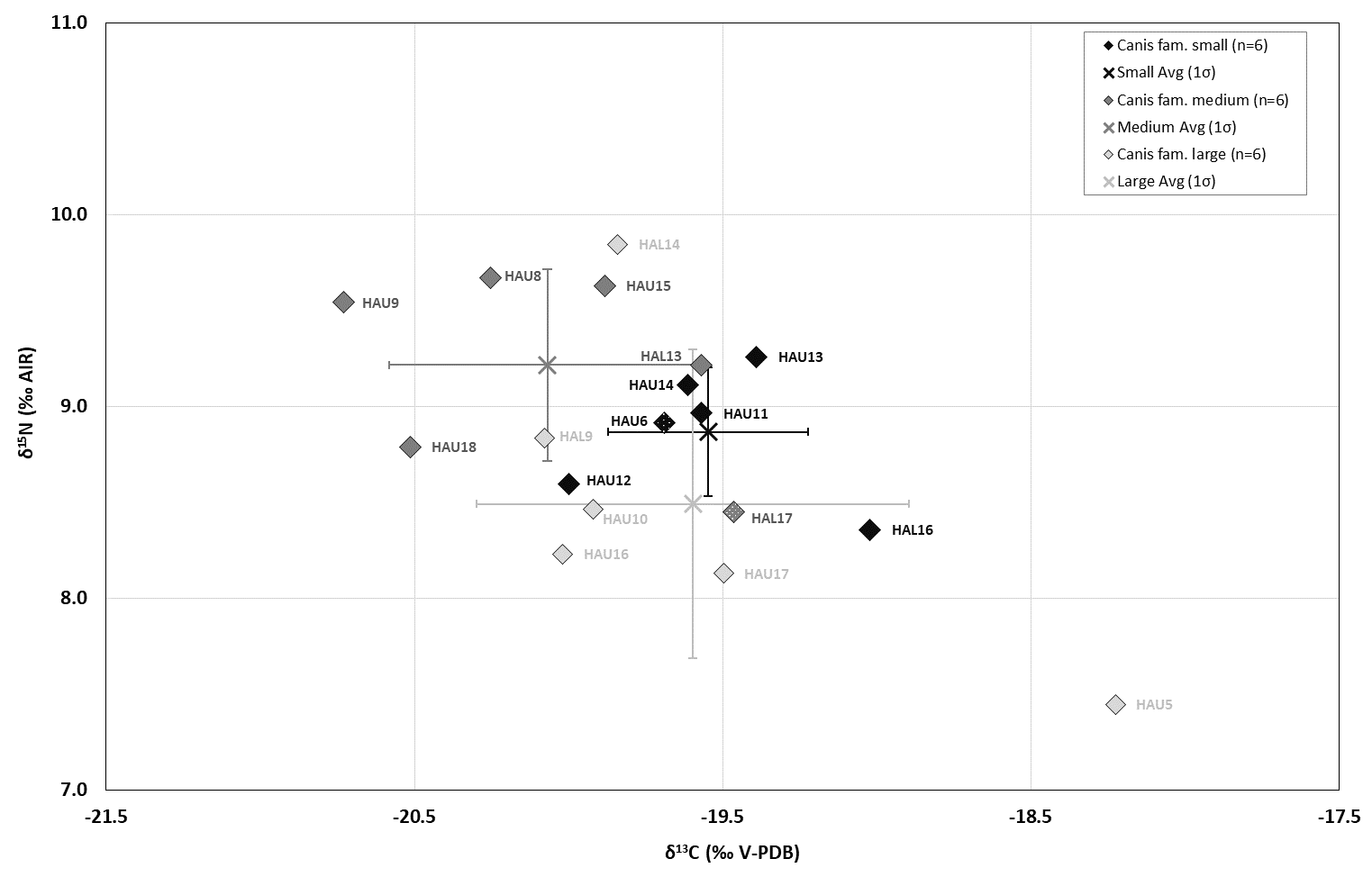


Figure S3. Stable carbon and nitrogen isotope compositions of 18 dogs from Augusta Raurica (16 adult animals (filled symbols), two juvenile dogs (dotted symbols)) according to size ranges (small (<39 cm), middle (40-59 cm), and large (>60 cm)). Dog ID’s as listed in Table S1.


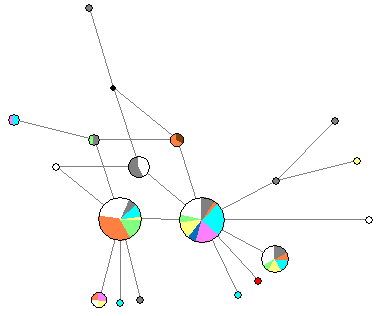

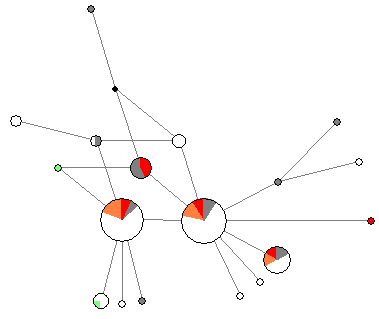

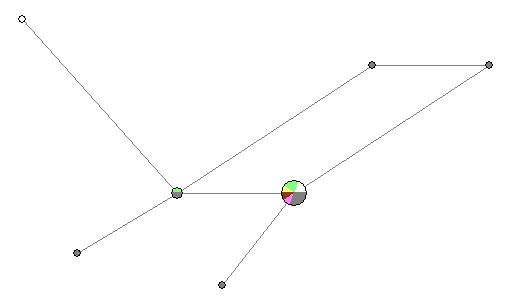

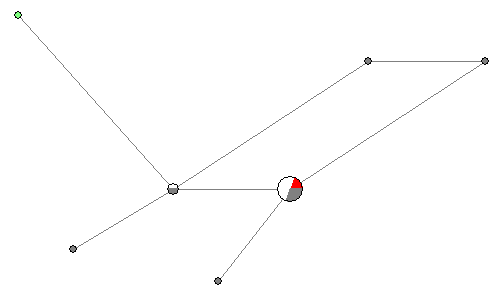

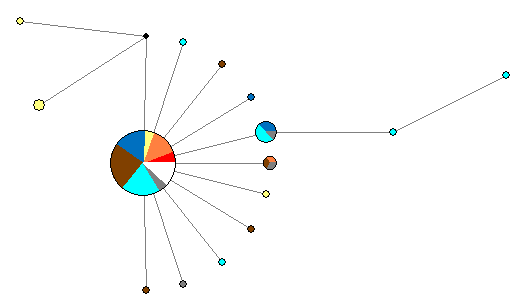

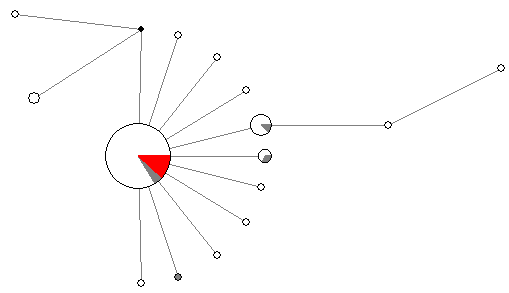

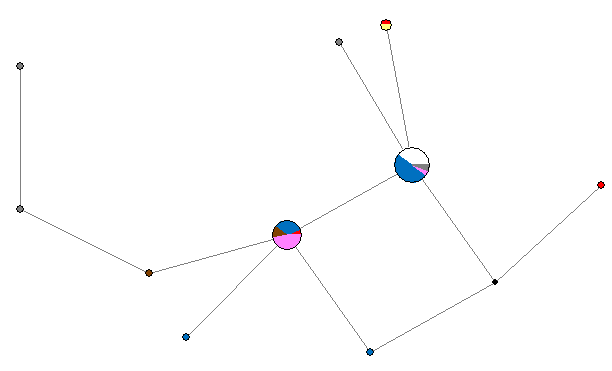

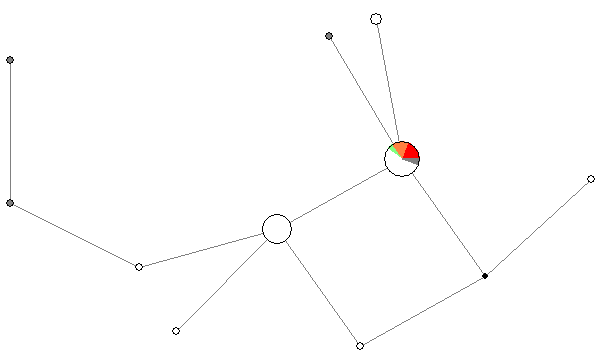


RS-a4

RS-a2

RS-a1

RS-a3

RS-a5

RS-a4

RS-a2

RS-a3

RS-a1

RS-a5

RS-b1

RS-b1

RS-c1

RS-c1

RS-d1

RS-d1

D

C

B

A

Roman + modern

Pre-Roman + modern

Switzerland

Iberia/Morocco

Central Western Europe

Southwest and Central Asia

Western Northern Europe/Russia

Bulgaria

Italy

Southeast Europe

modern

5

10

30

60

◦

1

Figure S4. Median-Joining Network of pre-Roman (n=178), Roman (n=45) and modern (n=34) mtDNA sequences (44 bp). Haplogroups A, B, C and D follow nomenclature of Duleba et al. 2015^41^ based on diagnostic polymorphic sites within D-loop region. Eight different haplotypes were found in Roman Switzerland (see designation of circles). Median vector () denotes hypothetical haplotype. Arrows indicate continuity of haplotypes from preceding time period. Details as listed in Table S2.


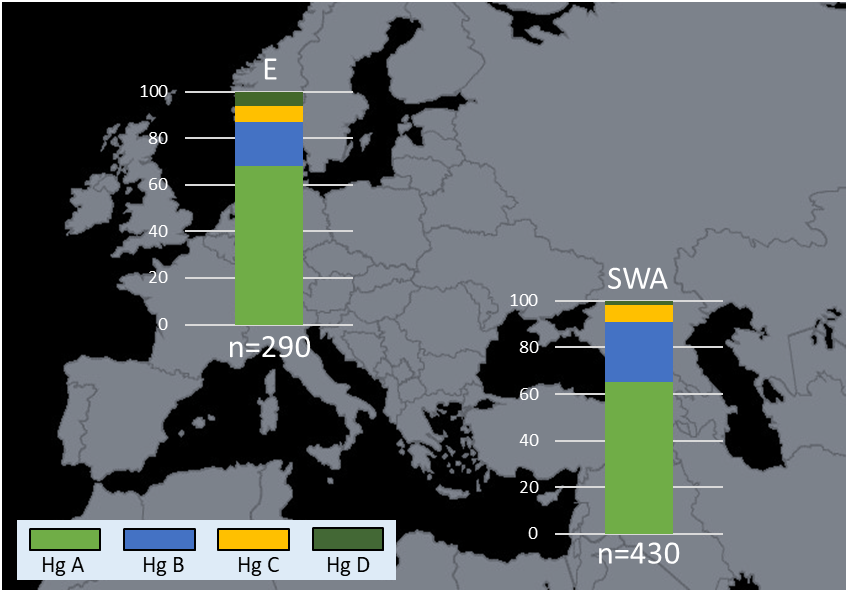


Figure S5. Haplogroup (Hg) A, B, C and D frequencies (0-100 %, y-axis) in modern dogs of Europe (E) and Southwest Asia (SWA). Free available map downloaded from slidelizard.com (<https://slidelizard.com/en/blog/powerpoint-world-map>) and edited using Microsoft Office 2016.


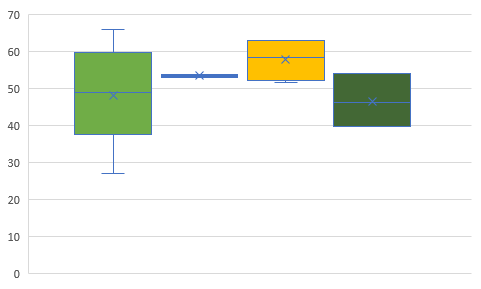


withers height (cm)

A B C D

n=6 n=2 n=4 n=3

Figure S6. Size distribution according to haplogroups A, B, C and D. Only adult dogs from the two wells of Augusta Raurica are considered (n=15).


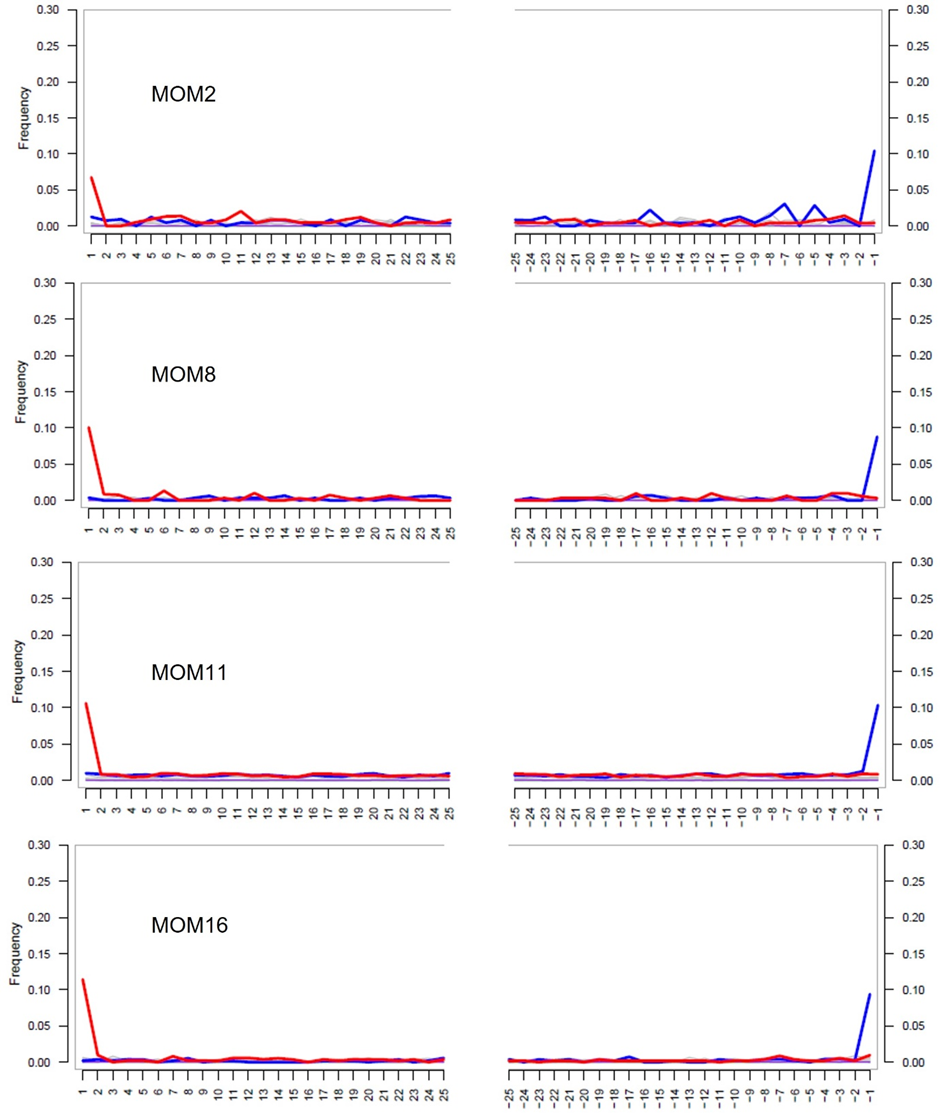


HAL2 1700-1650 (CH)

HAL11 1700-1650 (CH)

HAL8 1700-1650 (CH)

HAL16 1700-1650 (CH)

Figure S7. aDNA damage patterns for mitogenomes HAL2 1700-1650 (CH),

HAL8 1700-1650 (CH), HAL11 1700-1650 (CH) and HAL16 1700-1650 (CH) showing the expected degradation patterns, increased C > T and G > A substitutions at the 5´ and 3´ends of the reads.

Table S1. Overview and details of samples used in this study: archaeology, metrics, DNA and stable isotope information.

Excel file available online

Table S2. Used sequences in Median-Joining Network analysis.

Excel file available online

Table S3. Number of generated and clipped and merged reads for each sample

| Sample | Generated reads | Clipped and merged reads | Endogenous DNA content |
| --- | --- | --- | --- |
| HAL1 | 76685180 | 39492762 | 0,0002% |
| HAL2 | 121533948 | 61843069 | 25,09% |
| HAL3 | 166341286 | 87496248 | 3,4% |
| HAL4 | 63476562 | 32791453 | 2,7% |
| HAL5 | 107971444 | 57041912 | 0,49% |
| HAL6 | 57607950 | 30470313 | 10,7% |
| HAL7 | 73644790 | 39414688 | 0,81% |
| HAL8 | 127694014 | 68838417 | 1,71% |
| HAL11 | 149698456 | 80112381 | 4,63% |
| HAL12 | 65234646 | 34276134 | 1,07% |
| HAL13 | 86619292 | 46499474 | 0,55% |
| HAL14 | 154018488 | 77081948 | 5,37% |
| HAL15 | 139326000 | 69289581 | 1,6% |
| HAL16 | 97580960 | 48652113 | 7,77% |

Table S4. Basic mapping statistics after alignment against the *Canis lupus familiaris* mitochondrial genome (NC_002008.4) for all 14 samples

| Sample | Mapping reads | Coverage % | Meandepth |
| --- | --- | --- | --- |
| HAL1 | 82 | 23,8716 | 0,268727 |
| HAL2 | 1163 | 97,7103 | 4,35679 |
| HAL3 | 749 | 94,0097 | 2,79255 |
| HAL4 | 569 | 86,2438 | 2,0492 |
| HAL5 | 107 | 30,5853 | 0,354098 |
| HAL6 | 182 | 43,361 | 0,573026 |
| HAL7 | 330 | 70,2577 | 1,2218 |
| HAL8 | 1578 | 99,181 | 5,74502 |
| HAL11 | 15356 | 99,9701 | 54,5679 |
| HAL12 | 171 | 45,9138 | 0,638967 |
| HAL13 | 127 | 34,1723 | 0,440067 |
| HAL14 | 533 | 78,5497 | 1,59748 |
| HAL15 | 871 | 94,9662 | 3,055 |
| HAL16 | 2692 | 99,9223 | 9,95122 |

Table S5. Detailed mapping statistics for the four samples (HAL2, HAL8, HAL11 and HAL16) that exhibited good coverage of the reference genome

| Sample | Mitogenome | Coverage % >=1X | Coverage % >=2X | Coverage % >=3X | Meandepth |
| --- | --- | --- | --- | --- | --- |
| HAL2 | HAL2 1700-1650 (CH) | 97.71 | 90.56 | 78.18 | 4,3567 |
| HAL8 | HAL8 1700-1650 (CH) | 99.18 | 96.32 | 89.36 | 5,7450 |
| HAL11 | HAL2 1700-1650 (CH) | 99.97 | 99.96 | 99.94 | 54,5679 |
| HAL16 | HAL2 1700-1650 (CH) | 99.92 | 99.82 | 99.00 | 9,9512 |

Table S6. List of sequences used for maximum-likelyhood tree construction

Excel file available online

**References**

1 Berger, L. *Führer durch Augusta Raurica*. (Schwabe Verlag, 2012).

2 Bossart, J. *et al.* Zur Einwohnerzahl von Augusta Raurica. *Jahresberichte aus Augst und Kaiseraugst* **27**, 67-108 (2006).

3 Deschler-Erb, S., Stopp, B. & Vandorpe, P. Big Data - 65 Jahre archäobiologische Forschungen in Augusta Raurica *Jahresberichte aus Augst und Kaiseraugst 42*, 293-368 (2021).

4 Mráz, M. Bärenhatzen und Hundeplagen - Tierknochen aus dem Sodbrunnen MR12 in der Region 17C der Unterstadt von Augusta Raurica. *Jahresberichte aus Augst und Kaiseraugst* **39**, 143-182 (2019).

5 Schmid, D., Peter, M. & Deschler-Erb, S. in *L'Empire Romain en Mutation - Répercussions sur les villes dans la deuxième moitié du 3e siècle. Colloque International / Das römische Reich im Umbruch - Auswirkungen auf die Städte in der zweiten Hälfte des 3. Jahrhunderts. Internationales Kolloquium, Bern/August (Schweiz), 3.-5.12.2009. Archéologie et Histoire Romaine* Vol. 20 (eds Regula Schatzmann & Stefanie Martin-Kilcher) 125-132 (Éditions Monique Mergoil 2011).

6 Müller, U., Grolimund, L. & Saner, C. Ausgrabungen in Kaiseraugst im Jahre 2003. *Jahresberichte aus Augst und Kaiseraugst* **25**, 179-196 (2004).

7 Pfäffli, B. & Rychener, J. in *Jahresberichte aus Augst und Kaiseraugst* Vol. 26 (ed Römerstadt Augusta Raurica) 71-86 (Römermuseum Augst, 2005).

8 Breuer, G. Die Tierknochenfunde aus zwei Latrinengruben des 1. Jahrhunderts in Augst (Grabung 1991.65) unter besonderer Berücksichtigung der Hundeskelette. *Jahresberichte aus Augst und Kaiseraugst* **13**, 177-196 (1992).

9 Trumm, J. Vindonissa: Stand der Erforschung. Teil I, Vorgeschichte, keltische zeit und der militärische Komplex. *Jahresbericht der Gesellschaft Pro Vindonissa 2010*, 37-53 (2011).

10 Deschler-Erb, S. & Akeret, Ö. Archäobiologische Forschungen zum römischen Legionslager von Vindonissa und seinem Umland: Status quo und Potenzial. *Jahresbericht Gesellschaft Pro Vindonissa 2010*, 13-36 (2011).

11 Trumm, J., Akeret, Ö. & Flück, M. *Am Südtor von Vindonissa: die Steinbauten der Grabung Windisch-Spillmannwiese 2003-2006 (V. 003.1) im Süden des Legionslagers*. (Kantonsarchäologie Aargau, 2013a).

12 Flück, H. *Vor den Toren von Vindonissa. Wohnen und Arbeiten in einem Handwerkerquartier in den Canabae des Legionslagers (Windisch Zivilsiedlung West 2006-2008)* Vol. 23 (Veröffentlichungen der Gesellschaft Pro Vindonissa, 2017).

13 Trumm, J., Brogli, R. F., Frei-Stolba, R. & Kahlau, T. Bestattet und begraben: ein neu entdecktes römisches Gräberfeld in Vindonissa. *Archäologie der Schweiz* **36**, 26-33 (2013b).

14 Deschler-Erb, S. & Stopp, B. in *Am Südtor von Vindonissa. Die Steinbauten der Grabung Windisch-Spillmannwiese 2003-2006 (V.003.1) im Süden des Legionslagers.* Vol. 22 (eds J. Trumm & M. Flück) 463-477 (Veröffentlichungen der Gesellschaft Pro Vindonissa, 2013).

15 Kaltenthaler, D. *et al.* *OssoBook v20.1*. (2022).

16 Clark, K. M. The later prehistoric and protohistoric dog : the emergence of canine diversity. *Archaeozoologia* **7**, 9-32 (1995).

17 Koudelka, F. Das Verhältniss der Ossa longa zur Skelethöhe bei den Säugetieren. *Verhandlungen des naturforschenden Vereins Brünn* **24**, 127-153 (1885).

18 Club, A. K. *The Complete Dog Book*. 790 (Howell Book House, 1998).

19 Parker Heidi, G. *et al.* An Expressed Fgf4 Retrogene Is Associated with Breed-Defining Chondrodysplasia in Domestic Dogs. *Science* **325**, 995-998 (2009).

20 Brown, E. A. *et al.* FGF4 retrogene on CFA12 is responsible for chondrodystrophy and intervertebral disc disease in dogs. *Proceedings of the National Academy of Sciences* **114**, 11476-11481 (2017).

21 Bartosiewicz, L. *Shuffling Nags, Lame Ducks. The Archaeology of Animal Disease*. (Oxbow, 2013).

22 Habermehl, K. H. *Die Altersbestimmung bei Haus- und Labortieren*. (Paul Parey, 1975).

23 Horard-Herbin, M. P. in *Dogs through time: An archaeological perspective* Vol. 889 (ed S. J. Crockford) 115-121 (BAR International Series, 2000).

24 Goodwin, E. A. & Ballard, W. B. Use of tooth cementum for age determination of gray wolves. *Journal of Wildlife Management* **49**, 313-316 (1985).

25 Mbizah, M. M., Steenkamp, G. & Groom, R. J. Evaluation of the Applicability of Different Age Determination Methods for Estimating Age of the Endangered African Wild Dog (*Lycaon Pictus*). *PLoS One* **11**, e0164676 (2016).

26 Longin, R. New Method of Collagen Extraction for Radiocarbon Dating. *Nature* **230**, 241-242 (1971).

27 Ambrose, S. H. Preparation and characterization of bone and tooth collagen for isotopic analysis. *Journal of Archaeological Science* **17**, 431-451 (1990).

28 Oelze, V. M. *et al.* Early Neolithic diet and animal husbandry: stable isotope evidence from three Linearbandkeramik (LBK) sites in Central Germany. *Journal of Archaeological Science* **38**, 270-279 (2011).

29 Knipper, C. *et al.* What is on the menu in a Celtic town? Iron Age diet reconstructed at Basel-Gasfabrik, Switzerland. *Archaeological and Anthropological Sciences* **9**, 1307-1326 (2017).

30 Prowse, T., Schwarcz, H. P., Saunders, S., Macchiarelli, R. & Bondioli, L. Isotopic paleodiet studies of skeletons from the Imperial Roman-age cemetery of Isola Sacra, Rome, Italy. *Journal of Archaeological Science* **31**, 259-272 (2004).

31 Jørkov, M. L. S., Jørgensen, L. & Lynnerup, N. Uniform diet in a diverse society. Revealing new dietary evidence of the Danish Roman Iron Age based on stable isotope analysis. *American Journal of Physical Anthropology* **143**, 523-533 (2010).

32 Redfern, R. C., Hamlin, C. & Athfield, N. B. Temporal changes in diet: a stable isotope analysis of late Iron Age and Roman Dorset, Britain. *Journal of Archaeological Science* **37**, 1149-1160 (2010).

33 Pate, F. D., Henneberg, R. J. & Henneberg, M. Stable Carbon and Nitrogen Isotope Evidence for Dietary Variability at Ancient Pompeii, Italy. *Mediterranean Archaeology & Archaeometry* **16**, 127-133 (2016).

34 Bourbou, C., Arenz, G., Dasen, V. & Lösch, S. Babes, bones, and isotopes: A stable isotope investigation on nonadults from Aventicum, Roman Switzerland (first–third century CE). *International journal of osteoarchaeology* **29**, 974-985 (2019).

35 Varano, S. *et al.* The edge of the Empire: diet characterization of medieval Rome through stable isotope analysis. *Archaeological and Anthropological Sciences* **12**, 1-16 (2020).

36 Granado, J. D. *et al.* The mules that are not mules - metrics, morphology, archaeogenomics and mtDNA d-loop diversity in equids from Roman Switzerland. *Journal of Archaeological Science* **123**, 105253 (2020).

37 Frantz, L. A. *et al.* Genomic and archaeological evidence suggest a dual origin of domestic dogs. *Science* **352**, 1228-1231 (2016).

38 Kim, K. S., Lee, S. E., Jeong, H. W. & Ha, J. H. The complete nucleotide sequence of the domestic dog (*Canis familiaris*) mitochondrial genome. *Molecular Phylogenetics and Evolution* **10**, 210-220 (1998).

39 Verginelli, F. *et al.* Mitochondrial DNA from prehistoric canids highlights relationships between dogs and South-East European wolves. *Molecular Biology and Evolution* **22**, 2541-2551 (2005).

40 Excoffier, L. & Lischer, H. E. Arlequin suite ver 3.5: a new series of programs to perform population genetics analyses under Linux and Windows. *Molecular ecology resources* **10**, 564-567 (2010).

41 Duleba, A., Skonieczna, K., Bogdanowicz, W., Malyarchuk, B. & Grzybowski, T. Complete mitochondrial genome database and standardized classification system for *Canis lupus familiaris*. *Forensic Science International: Genetics* **19**, 123-129 (2015).

42 Bandelt, H.-J., Macaulay, V. & Richards, M. Median Networks: Speedy Construction and Greedy Reduction, One Simulation, and Two Case Studies from Human mtDNA. *Molecular Phylogenetics and Evolution* **16**, 8-28 (2000).

43 Pires, A. E. *et al.* Roman dogs from the Iberian Peninsula and the Maghreb – A glimpse into their morphology and genetics. *Quaternary International* **471**, 132-146 (2018).

44 Yankova, I. *et al.* Evidence for Early European Neolithic Dog Dispersal: New Data on Southeastern European Subfossil Dogs from the Prehistoric and Antiquity Ages. *Genes* **10**, 757 (2019).

45 Pang, J.-F. *et al.* mtDNA Data Indicate a Single Origin for Dogs South of Yangtze River, Less Than 16,300 Years Ago, from Numerous Wolves. *Molecular Biology and Evolution* **26**, 2849-2864 (2009).

46 Webb, K. M. & Allard, M. W. Mitochondrial Genome DNA Analysis of the Domestic Dog: Identifying Informative SNPs Outside of the Control Region. *Journal of Forensic Sciences* **54**, 275-288 (2009).

47 Savolainen, P., Zhang, Y.-p., Luo, J., Lundeberg, J. & Leitner, T. Genetic Evidence for an East Asian Origin of Domestic Dogs. *Science* **298**, 1610 (2002).

48 Strakova, A. *et al.* Mitochondrial genetic diversity, selection and recombination in a canine transmissible cancer. *eLife* **5**, e14552 (2016).

49 Björnerfeldt, S., Webster, M. T. & Vilà, C. Relaxation of selective constraint on dog mitochondrial DNA following domestication. *Genome research* **16**, 990-994 (2006).

50 Angleby, H. *et al.* Forensic Informativity of ~3000 bp of Coding Sequence of Domestic Dog mtDNA. *Journal of Forensic Sciences* **59**, 898-908 (2014).

51 Scheible, M. K. R., Straughan, D. J., Burnham-Curtis, M. K. & Meiklejohn, K. A. Using hybridization capture to obtain mitochondrial genomes from forensically relevant North American canids: Assessing sequence variation for species identification. *Forensic Science International: Animals and Environments* **1**, 100018 (2021).

52 Imes, D. L., Wictum, E. J., Allard, M. W. & Sacks, B. N. Identification of single nucleotide polymorphisms within the mtDNA genome of the domestic dog to discriminate individuals with common HVI haplotypes. Forensic Sci Int Genet 6, 630-639, doi:10.1016/j.fsigen.2012.02.004 (2012).

53 Marinov, M., Teofanova, D., Gadjev, D., Radoslavov, G. & Hristov, P. Mitochondrial diversity of Bulgarian native dogs suggests dual phylogenetic origin. *PeerJ* **6**, e5060 (2018).

54 Koupadi, K. *et al.* Population Dynamics in Italian Canids between the Late Pleistocene and Bronze Age. *Genes* **11**, 1409 (2020).

55 Ciucani, M. M. *et al.* Old wild wolves: ancient DNA survey unveils population dynamics in Late Pleistocene and Holocene Italian remains. *Peerj* **7**, e6424 (2019).

56 Boschin, F. *et al.* The first evidence for Late Pleistocene dogs in Italy. *Sci Rep* **10**, 13313 (2020).

57 Loog, L. *et al.* Ancient DNA suggests modern wolves trace their origin to a Late Pleistocene expansion from Beringia. *Mol Ecol*, 1-15 (2019).

58 Thalmann, O. *et al.* Complete Mitochondrial Genomes of Ancient Canids Suggest a European Origin of Domestic Dogs. *Science* **342**, 871 (2013).

59 Pionnier-Capitan, M. *Thesis*. (École Normale Supérieure de Lyon, Lyon, France 2010).

60 Pires, A. E. *et al.* The curious case of the Mesolithic Iberian dogs: An archaeogenetic study. *Journal of Archaeological Science* **105**, 116-129 (2019).

61 Deguilloux, M. F., Moquel, J., Pemonge, M. H. & Colombeau, G. Ancient DNA supports lineage replacement in European dog gene pool: insight into Neolithic southeast France. *Journal of Archaeological Science* **36**, 513-519 (2009).

60 Botigué, L. R. *et al.* Ancient European dog genomes reveal continuity since the Early Neolithic. *Nature Communications* **8**, 16082 (2017).

63 Bergström, A. *et al.* Origins and genetic legacy of prehistoric dogs. *Science* **370**, 557-564 (2020).

64 Druzhkova, A. S. *et al.* Ancient DNA analysis affirms the canid from Altai as a primitive dog. *PLoS One* **8**, e57754 (2013).

65 Malmström, H., Stora, J., Dalen, L., Holmlund, G. & Gotherstrom, A. Extensive human DNA contamination in extracts from ancient dog bones and teeth. *Mol Biol Evol* **22**, 2040-2047 (2005).

66 Krause-Kyora, B. *et al.* Ancient DNA study reveals HLA susceptibility locus for leprosy in medieval Europeans. *Nature Communications* **9**, 1569 (2018).

67 Susat, J. *et al.* *Yersinia pestis* strains from Latvia show depletion of the pla virulence gene at the end of the second plague pandemic. *Sci Rep* **10**, 14628 (2020).

68 Li, H. & Durbin, R. Fast and accurate short read alignment with Burrows-Wheeler transform. *Bioinformatics* **25**, 1754-1760 (2009).

69 Peltzer, A. et al. EAGER: efficient ancient genome reconstruction. Genome Biol 17, 60, doi:10.1186/s13059-016-0918-z (2016).

70 Jónsson, H., Ginolhac, A., Schubert, M., Johnson, P. L. & Orlando, L. mapDamage2.0: fast approximate Bayesian estimates of ancient DNA damage parameters. *Bioinformatics* **29**, 1682-1684 (2013).

71 McKenna, A. *et al.* The Genome Analysis Toolkit: a MapReduce framework for analyzing next-generation DNA sequencing data. *Genome Research* **20**, 1297-1303 (2010).

72 Matsumura, S., Inoshima, Y. & Ishiguro, N. Reconstructing the colonization history of lost wolf lineages by the analysis of the mitochondrial genome. *Molecular phylogenetics and evolution* **80**, 105-112 (2014).

73 Baranowska, I. *et al.* Sensory Ataxic Neuropathy in Golden Retriever Dogs Is Caused by a Deletion in the Mitochondrial tRNATyr Gene. *PLOS Genetics* **5**, e1000499 (2009).

74 Jia, Q.-H., Liu, H.-B., Gu, X.-L., Li, J.-F. & Liu, Y.-L. Complete mitochondrial genome of a German Shepherd (*Canis lupus familiaris* breed German Shepherd) provides insights into genome-wide sequence variations. *Mitochondrial DNA Part A* **27**, 118-119 (2016).

75 Hao, Z., Zhang, Q. & Qu, B. The complete mitochondrial genome of the Chinese indigenous dog. *Mitochondrial DNA Part A* **27**, 88-89 (2016).

76 Tian, J., Zhou, J. & Xiao, M.-F. The complete mitochondrial genome of Belgium Malinois (*Canis*; Canidae). *Mitochondrial DNA Part A* **27**, 171-172 (2016).

77 Verscheure, S., Backeljau, T. & Desmyter, S. Dog mitochondrial genome sequencing to enhance dog mtDNA discrimination power in forensic casework. *Forensic Science International: Genetics* **12**, 60-68 (2014).

78 Ameen, C. *et al.* Specialized sledge dogs accompanied Inuit dispersal across the North American Arctic. *Proc Biol Sci* **286**, 20191929 (2019).

79 Katoh, K., Misawa, K., Kuma, K. & Miyata, T. MAFFT: a novel method for rapid multiple sequence alignment based on fast Fourier transform. *Nucleic Acids Res* **30**, 3059-3066 (2002).

80 Talavera, G. & Castresana, J. Improvement of phylogenies after removing divergent and ambiguously aligned blocks from protein sequence alignments. *Syst Biol* **56**, 564-577 (2007).

81 Stamatakis, A. RAxML version 8: a tool for phylogenetic analysis and post-analysis of large phylogenies. *Bioinformatics* **30**, 1312-1313 (2014).

82 Rambaut, A. FigTree [Internet]. Available from: http://tree.bio.ed.ac.uk/software/figtree/.

83 Gerling, C. Neither fish nor fowl. Isotopic evidence of a plant-based diet in (captive?) brown bears from Roman Augusta Raurica, Switzerland. Anthropozoologica 58, 59-72 (2023).
